# Normal autonomic neurophysiology of postural orthostatic tachycardia and recommended physiological assessments in postural orthostatic tachycardia syndrome

**DOI:** 10.14814/phy2.14465

**Published:** 2020-06-26

**Authors:** Peter Oketa‐Onyut Julu

**Affiliations:** ^1^ Clinical Research Centre William Harvey Heart Centre Barts and the London School of Medicine and Dentistry Charterhouse Square London UK

**Keywords:** baroreflex, brainstem, cardiac vagal tone, cardiodepressor, chronotrophy, inotrophy, organotopic neurons, orthostasis, tachycardia, vascular resistance, vasodepressor, venous capacitance

## Abstract

The current surge of interest in postural orthostatic tachycardia syndrome commonly known as POTS requires good knowledge of the very complex physiology involved, but this is currently lacking. The often overlooked normal physiology of orthostasis is reviewed including the definition of normal postural orthostatic tachycardia. An illustrated functional anatomy that embeds orthostatic tachycardia within the learned and skilful motor functions in the human population is presented. The four physiological phases of orthostasis and the role of tachycardia are described in a laboratory‐controlled and progressive orthostatic stress in normal human volunteers. Standardized surrogate measures of autonomic control were used to quantify the trigger level for excessive tachycardia and the minimum autonomic control required to sustain viable arterial blood pressure during severe orthostatic stress in normal human volunteers. Tachycardia during orthostasis is part of a “democratic” contribution by four cardiovascular parameters of which the chronotropic function of the heart is just one of the parameters contributing toward cardiovascular compensation. It is adjusted during orthostasis in proportion to contributions from the other three parameters, namely inotropic function of the heart, windkessel vascular resistance and venous vascular capacitance. The physiological effects of the two stressors during orthostasis, gravity and isometric contraction of skeletal muscles are reviewed. A model of how the four cardiovascular parameters are regulated during orthostasis to achieve proportionate contributions is proposed emphasizing the necessity to quantify individual contributions from all these four parameters. Any one or more of these parameters may be compromised due to disease requiring disproportionate contribution of the prevailing magnitude of orthostatic tachycardia in an individual. It therefore requires neurophysiological assessment of the autonomic regulation of all the four cardiovascular parameters to assess the condition fully. We recommend here some current and novel neurophysiological methods that use modern medical technology to quantify laboratory standardized surrogate measures of some of these cardiovascular parameters including central parasympathetic regulation in postural orthostatic tachycardia syndrome.

## Graphical abstract

A four‐parameter model of cardiovascular regulation, two each in the cardiac zone C and vascular zone D to explain the normal physiology of postural orthostatic tachycardia in the human population. The International Consensus definition of an autonomic disorder known as Postural Orthostatic Tachycardia Syndrome (POTS) conversely describes a natural and physiological cardiovascular compensation in Phase 2 of severe orthostatic stress. The functional anatomy of orthostasis shows that it is part of a learned skilful motor function in the human population including the associated tachycardia.

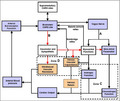

## 1 INTRODUCTION

### 1.1 The aim of this review

The current surge of interest in the disease known as Postural Orthostatic Tachycardia Syndrome or POTS has disenfranchized the natural and normal physiological status of cardiovascular compensations during mild and severe orthostatic stress. Although the disease POTS is a recognized dysautonomia, the whole phenomenon of postural orthostatic tachycardia has so far been turned into a popular disease due to lack of full knowledge of the complex but perfectly normal and natural physiology involved. The aim of this review is to restore the understanding of the natural and physiological basis of tachycardia during orthostasis (POT). This review will define the normal and natural POT in terms of a normal physiological phenomenon that can be reproduced in a normal human population in a physiology laboratory. The natural forces that exert the orthostatic stress that generates tachycardia in the human population are also presented. The functional arrangement of the neurons that generate tachycardia in POT in the human population (the functional anatomy) is presented for the first time in order to demonstrate the natural origin POT in humans. This review includes a four‐parameter model of cardiovascular compensation during orthostasis in humans and how they can be assessed in a clinical laboratory to investigate the disease POTS.

### 1.2 Definition of postural orthostatic tachycardia

#### 1.2.1 Origins of words

The phrase “Postural Orthostatic Tachycardia” refers to the quickening of heartbeats in response to a change in the position of the body, usually from recumbent to an upright position. The adjective postural has the Latin origin “*positura*” meaning position and the adjective orthostatic originates from two Greek words; “*orthos*” meaning upright and “*statos*” meaning standing. Hermann Lebert who was a physician introduced the term tachycardia in medicine in 1867. Tachycardia comes from two words of different origins, an English word “tachy” which means swift and the Greek word “*kardia*” which means the heart. Tachycardia literally means “swift heart”. Orthostasis is a verb meaning standing upright without translational movements, indicating clearly that the position of the subject is not shifting relative to the ground on which he or she is standing. It is very important to carefully define the phrase “postural orthostatic tachycardia" so that we understand fully that it actually refers to a natural and normal response of the heart to various and often numerous challenges to the human body during orthostasis. These challenges will be discussed later in this review.

#### 1.2.2 Sequence of events that defines postural orthostatic tachycardia

**FIGURE 1:**
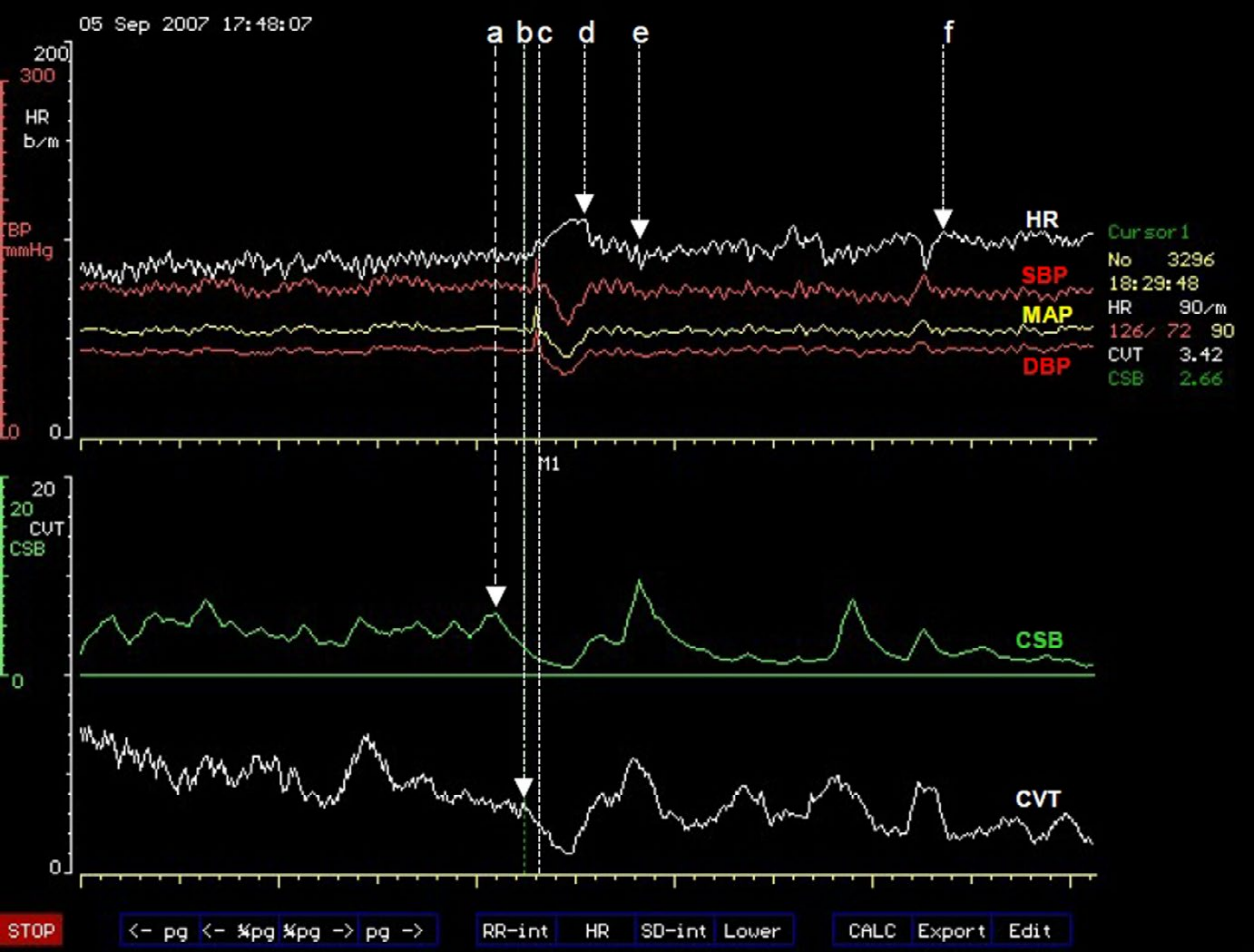
A computer screen capture from the NeuroScope displaying the following beat‐to‐beat traces of cardiovascular parameters. Heart rate (HR) expressed in beats.min^−1^; systolic (SBP), diastolic (DBP) and mean arterial blood pressures (MAP) measured in mmHg; cardiac sensitivity to baroreflex (CSB) measured in ms.mmHg^−1^ and cardiac vagal tone (CVT) measure in units of a linear vagal scale (LVS) (see text for descriptions and methods used to measure these cardiovascular parameters). The command to stand up was given at (a) triggering CSB withdrawal. CVT withdrawal started concurrently with a ramp tachycardia 10 s later than the “stand up” command at (b). The subject achieved an upright stance 8 s after the beginning of the ramp tachycardia at (c). The ramp tachycardia came to an abrupt end at (d) lasting for 19 s. The rebound bradycardia that followed ended at (e) and the HR recovered to a stable level in the upright stance at (f). Cardiovascular compensation with stable HR in the upright stance was achieved within 128 s (from c to f)

The heartbeat can start to quicken early during the thought of changing body position, even before the body begins to move (Figure 1). It is an indication that the heart receives cues from diverse sources to respond to orthostasis in addition to the body movements relative to the ground. The rate of heartbeats, commonly referred to as the heart rate, changes continuously during orthostasis; starting with the thought of righting the body in the erect posture and continues changing exceeding the time at which an upright posture is achieved to a period when the heart rate stabilizes at a rate faster than it was at the beginning of the process. We shall refer to this as early Phase 1 of orthostasis (Figure 1). The various magnitudes of heart rate changes at different stages of this early Phase 1 of orthostasis until it stabilizes in a fully compensated cardiovascular state are well known and are used for detection of abnormalities associated with autonomic dysfunctions (Neilson, Ewing, Campbell, & Clarke, 1978) sometimes referred to as dysautonomia. This is what makes it important to accurately define both the stages and phases of orthostasis together with the range of normal magnitudes of changes in heart rate during these phases in order to use them as clinical tools for detecting diseases. For example, the ratio of the durations of cardiac cycles measured as intervals of electrocardiographic R‐waves during the trough of the rebound bradycardia and the peak of the tachycardia in the early Phase 1 of orthostasis is used to assess autonomic functions and is called the 30:15 ratio (Neilson et al., 1978). This test is called 30:15 ratio simply because the trough of the rebound bradycardia usually comes at or near the 30th heartbeat while the peak of the ramp tachycardia comes at or around the 15th heartbeat after achievement of the upright stance during orthostasis (Figure 1).

The heart rate is expected to stabilize and remain within a known normal range that is higher than it was in supine position for as long as the person maintains an upright stance without translational movement. This is known as Postural Orthostatic Tachycardia and the complex but normal physiological processes involved in achieving this is the subject of this review. A person overwhelmed by the orthostatic challenge and develops neurological symptoms like poor balance or lack of spatial awareness, or cardiac symptoms like weakness or palpitations, is described as having poor or sometimes total lack of orthostatic tolerance. The extreme of orthostatic intolerance is postural syncope.

#### 1.2.3 Standardized diagnostic maneuvers

Diagnostic orthostatic challenge can be achieved by asking the patient to rise up to an erect posture starting from a supine position (Kahn, 1988). There is a longer delay between the initiation of this process and eventual achievement of an upright posture in this method and it is also associated with increased signal noise during the process of changing posture. There is, however, a modified diagnostic maneuver where the patient is asked to rise up while seated on a low stool. This has a similar cardiovascular effect but with little signal noise and very short delay between the initiation and achievement of the upright posture (Julu & Hondo, 1992). The third and most common method is achieved using a tilt table. The patient will lie limply on the tilt table while being tilted at various angles starting from a horizontal supine position to 60° recline or up to 90° upright posture depending on the wish of the clinician. This is discussed in more detail elsewhere. The physiological differences between these standardized diagnostic orthostatic challenges will become clearer later in this review.

### 1.3 Functional anatomy of postural orthostatic tachycardia

#### 1.3.1 Organotopic organization of neurons

##### Early lessons from animal studies

Ablation plus nonspecific near‐neuron stimulation techniques showed that there were distinctive tachycardia and bradycardia zones in the brainstem and were called tachycardia or bradycardia centers in those days (Alexander, 1946). The rostral part of medulla oblongata was largely excitatory and the caudal part was largely inhibitory according to the responses of blood pressure, heart rate, and sympathetic outflow recorded from peripheral nerves. The rostral medulla was therefore referred to as the "pressor" or “vaso‐active" area while the caudal part of medulla was referred to as “depressor” or vaso‐reflex area of the brainstem (Alexander, 1946).

###### Rostral ventrolateral medulla (RVLM)

**FIGURE 2:**
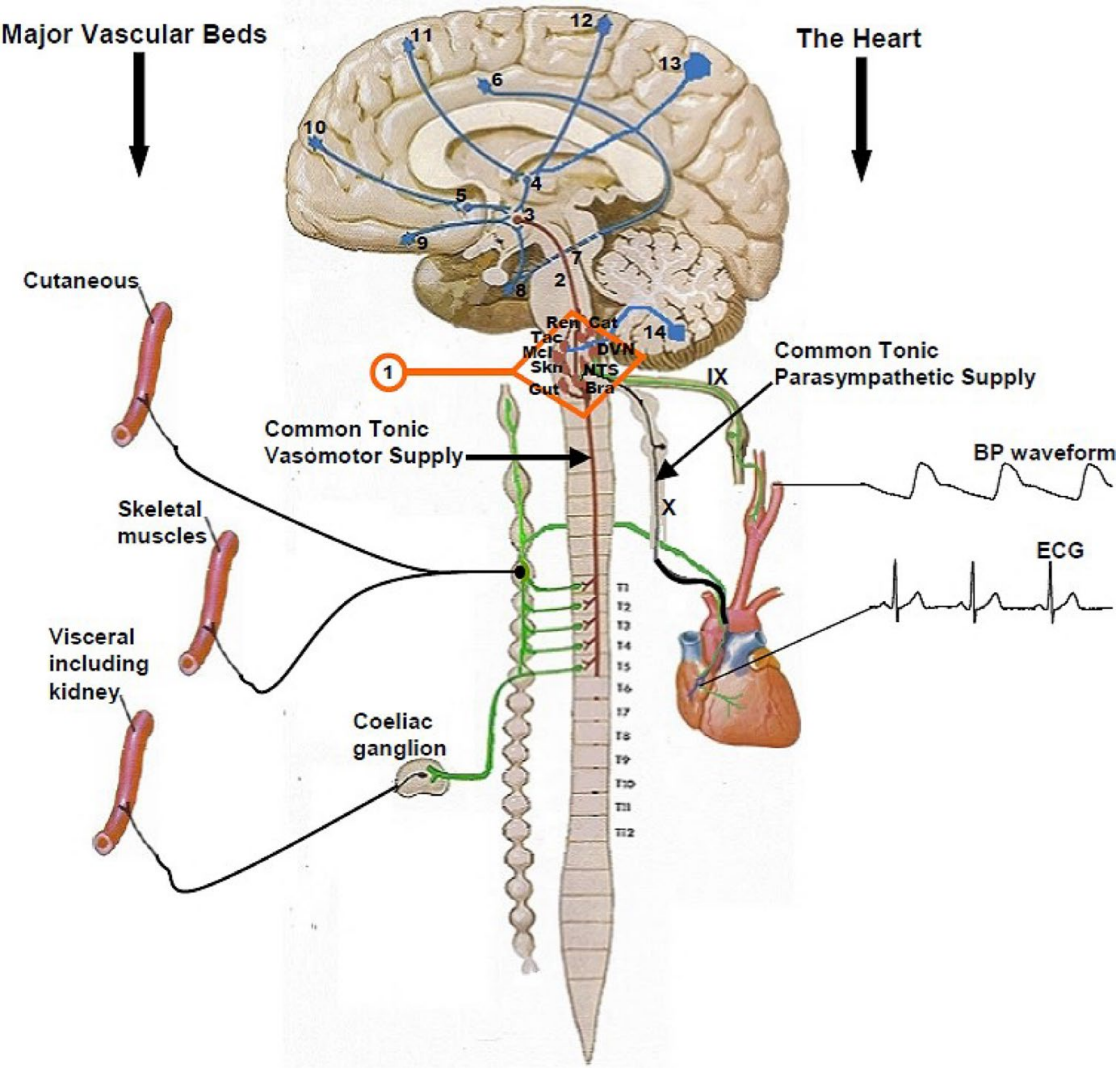
The mid sagittal section of the brain and the spinal cord used here for schematic illustration of the functional anatomy of postural orthostatic tachycardia. Electrocardiogram (ECG) and the arterial blood pressure waveform (BP waveform), both of which are generated by the heart, are the only two cardiovascular biometrics used to derive all the cardiovascular parameters displayed on the NeuroScope screen (see Figure 12 and main text for details). The Roman numerals show the ninth (IX) and tenth (X) cranial nerves. These are the most important nerves in the generation of central parasympathetic cardiovascular regulation and they provide the only tonic parasympathetic supply for the whole body in humans. These two cranial nerves communicate with the bradycardia area (Bra) in the caudal part of medulla oblongata and the tachycardia area (Tac) in the rostral part of the medulla (RVLM). Skeletal muscles vascular bed provides the most of the windkessel vascular resistance, while the visceral (or splanchnic) vascular bed provides the most of the venous vascular capacitance, but the sympathetic supply to the skin is largely suppressed during orthostasis (see main text for more details). These three vascular beds are important for arterial blood pressure defense during orthostasis. Animal studies show that there are organotopic neurons, which are discrete and separate groups of neurons in the RVLM that specifically regulate sympathetic supplies to the skin (Skn), skeletal muscles (Mcl), and the visceral (Gut) vascular beds. There are also organotopic neurons within the RVLM regulating catecholamine release (Cat) and sympathetic supply to the kidney (Ren). The Arabic numerals (1–14) within the brain and the brainstem show the central autonomic regulation sites (CARS) that have been described during functional magnetic resonance imaging (fMRI) and positron emission tomography (PET) studies of central autonomic functions. These are large sites in the brain that regulate several autonomic functions and some of the connections shown between the different CARS sites are based on animal studies (see main text for more details). The Arabic numerals (1–12) beside the spinal cord indicate the respective thoracic segments. The tonic vasomotor supplies to the three major vascular beds come out of the spinal between T1 and T5

Very early work in Carl Ludwig's laboratory had previously suggested that rostral medulla is a vasomotor center with automaticity with possible reflexogenic input (Seller, 1996). The development of microelectrodes that allow recording signals from single nerve fibers greatly improved our understanding of the organization of neurons in the brain, learning mainly from animal studies (Dampney, Blessing, & Tan, 1988). Glass tube microelectrodes facilitated microinjection of various known neuronal stimulants and enabled differential activation of neurons within the same area in the brain. This technique enabled categorization of neurons according to the neurotransmitters they use for communication (Boscan, Allen, & Paton, 2001; Sasaki & Dampney, 1990). Topographic arrangement of neurons in the brainstem controlling distinct and specific vascular beds has been described in the subretrofacial (SRF) region of the medullo‐pontine junction (Dampney & McAllen, 1988; McAllen & Dampney, 1990). The old vasomotor area is currently known as the rostral ventrolateral medulla (RVLM) where tonically active pre‐sympathetic neurons are situated (Guyenet, Haselton, & Sun, 1989). It is also now well established in animals that these pre‐sympathetic neurons are topographically organized according to their target‐organs in various vascular beds independent of the somatic map (McAllen & Dampney, 1990). The topography of the pre‐sympathetic neurons controlling various independent target‐organs that has so far been described in the RVLM in animal studies are depicted in the human brainstem in Figure 2. We shall in this review describe the topographic organization of pre‐sympathetic neurons in the RVLM as "Organotopic” organization meaning that the neurons are organized according to the target‐organs they control independently of the somatic map or which side of the body. The importance of the central autonomic regulation sites (CARS) shown in Figure 2 will become clearer later on in this review.

Takeaway points

1. The topographic arrangement of neurons in the RVLM is according to the target‐organs or some preset vascular beds they control and this is independent of the sides of the body whether left or right and we call this organotopic organization of neurons.
2. These organotopic neurons control their target‐organs through a common tonic vasomotor and sympathetic pathway (Figure 2) descending from the RVLM through the spinal cord to the multiple target‐organs they regulate in the body.
3. The tonic vasomotor supply to blood vessels in the body leaves the spinal cord at every segment starting from thoracic segment number one (T1) and it completes its exit out of the spinal cord at the level of thoracic segment number five (T5) in human (Nathan & Smith, 1987) and (Figure 2).
4. A central autonomic regulation site (CARS) is a distinct area of the brain that regulates several functions of the autonomic nervous system. For example, the RVLM is a discrete area in the brainstem that regulates several autonomic functions and it is situated in a CARS site labelled as number 1 just for identification purposes only in this review. More of these regulatory sites and their identification numbers are shown in Figure 2.

###### Caudal part of medulla oblongata

Functional organization of neurons in the caudal part of the medulla that was previously described as the bradycardia center is fully reviewed elsewhere (Gourine, Machhada, Trapp, & Spyer, 2016). The ventrolateral part of caudal medulla oblongata (cVLM) contains a network of neurons situated in separate and discrete nuclei in the caudal part of the brainstem. The arterial baroreceptor input through the ninth cranial nerve (IX) into the part of the brainstem depicted as bradycardia center in Figure 2 has first order synapses in the nucleus of tractus solitarius (NTS) (Lipski, McAllen, & Spyer, 1975). These baroreflex neurons are located in the medial nuclei of NTS in animals (Czachurski, Lackner, Ockert, & Seller, 1982) but the arrangement and architecture of neurons in the NTS are similar in human (McRitchie & Törk, 1993). The baroreflex neurons in the NTS project to neurons situated both in the RVLM and to a group of neurons located in the caudal part of nucleus ambiguus (NA) in the cVLM (Schreihofer & Guyenet, 2002). These are known as the cardiac vagal preganglionic neurons (cVPN). These cVPN in the caudal part of NA are the major source of the tonic parasympathetic negative chronotropic efferent to the heart and they have large myelinated B‐fiber axons projecting to the nodal tissues of the heart (McAllen & Spyer, 1976). The cVPN of NA have tonic activities, which mainly have cardiac pulse rhythms but they also have and do respond to respiratory rhythms (Gilbey, Jordan, Richter, & Spyer, 1984). There is another independent source of tonic parasympathetic efferent to the heart originating from the caudal part of dorsal vagal motor nucleus (DVMN). The cVPN situated in the caudal part of DVMN have tonic rhythms that do not respond to baroreflex, respiration or chemoreceptor stimulations (Jones, Wang, & Jordan, 1998). These cVPN of DVMN have pacemaker‐like activity with baseline firing rate between 0.5 and 6 Hz (Ballanyi, Doutheil, & Brockhaus, 1996), and they have slow unmyelinated C‐fiber axons projecting to the heart (Ford, Bennett, Kidd, & McWilliam, 1990). Stimulation of the left vagus nerve trunk has negative inotropic effect in human, causing load‐independent reduction of ventricular contractility as measured by the end‐systolic elastance (Lewis et al., 2001). The negative inotropic effect of stimulating the left vagus nerve trunk is blocked by the muscarinic antagonist glycopyrronium (Lewis et al., 2001). Since intra‐coronary administration of atropine enhances dobutamine‐induced inotropic responses of the left ventricle without affecting heart rate in human, but this effect is lost in denervated transplanted hearts (Landzberg, Parker, Gauthier, & Colucci, 1994), it is further evidence that there is a tonic parasympathetic restraint of the inotropic function of the heart in human coming from the cVPN of DVMN. This tonic parasympathetic ventricular restraint also regulates ventricular excitability as measured by effective refractory period, the threshold of triggered ventricular tachycardia and QT interval prolongation (Machhada et al., 2015, 2016). The cVPN of DVMN are critically important for the recruitment of innate mechanisms of inter‐organ protection underlying the phenomenon of remote ischemic conditioning (Mastitskaya et al., 2012) in which the heart is protected from myocardial ischemia and reperfusion injury by episodes of ischemia and reperfusion injuries in other tissues elsewhere in the body remote from the heart. The DVMN receives numerous modulatory inputs through the tenth cranial nerve (X, Figure 2) shown as vagal afferents, some of which has subdiaphragmatic origin, but all pass via the NTS to DVMN (Powley, 2000). There are also projections from hypothalamic nuclei in CARS site 3 and other forebrain areas like CARS sites 9 and 8 with modulatory inputs from the periaqueductal gray (PAG) matter in CARS site 7 (Figure 2) through the descending pathway in CARS site 2 directly to the DVMN at the caudal medulla schematically depicted as bradycardia area (Figure 2). These rich modulatory inputs are shared between the cVPN including those in the caudal part of DVMN (Powley, 2000). The supramedullary modulation of cVPN can be used to reduce the extent of lethal ischemia and reperfusion injury of the heart either via recruitment of direct cardiac projections with consequent myocardial acetylcholine release (Mastitskaya et al., 2012) or via triggering the production and release into the systemic circulation of certain gut hormones thought to have cardioprotective properties (Mastitskaya et al., 2016). Experimental evidence available suggests that the cardioprotective gut hormone(s) are released by the visceral organ(s) innervated by the posterior gastric branch of the vagus nerve and is abolished by cutting this nerve (Mastitskaya et al., 2016) showing the significance of DVMN in this process. There are therefore two tonic parasympathetic outputs originating from two different types of cVPN in the caudal part of the brainstem destined to the heart through a common pathway, which is the vagus nerve (Figure 2). One tonic parasympathetic output controls the chronotropic functions of the heart while the other tonic parasympathetic output controls the inotropic and excitability functions of the myocardium.

Takeaway points

1. A network of three discrete nuclei in the cVLM in CARS site 1 forms the only central source of tonic parasympathetic supply for the whole body. These nuclei are NTS, caudal parts of NA and DVMN.
2. The vagus nerve provides a common and the only pathway for two different types of outputs of tonic parasympathetic supplies from the brainstem to peripheral organs.
3. There are three types of rhythms that can be used to distinguish the origins of the two types of tonic central parasympathetic activities. The central parasympathetic tone with pacemaker‐like rhythm varying in frequencies between 0.5 and 6 Hz, and does not respond to respiratory chemoreceptor stimulation, cardiac pulsation, nor breathing rhythm, is generated by cVPN in DVMN. The central parasympathetic tone with inherent cardiac pulse and breathing rhythms and responds to respiratory chemoreceptor stimulation and also responds to voluntary changes in breathing rhythms is generated by cVPN in NA.

##### Lessons from functional magnetic resonance images (fMRI) studies in human

Invention of magnetic resonance imaging (MRI) of the brain that uses blood oxygen level dependent (BOLD) signal intensity (Ogawa, Lee, Nayak, & Glynn, 1990) has facilitated acquisition of useful and real‐time functional images (fMRI) of the human brain in three dimensions. The BOLD signal intensity can therefore be correlated with different brain functions being investigated in real‐time during various tasks. This has been used to investigate the brain areas regulating sympathetic activities at rest and during various maneuvers commonly deployed to challenge the autonomic nervous system during clinical examinations.

###### Baseline regulation of skeletal muscle sympathetic activity in supine position

Baseline brain control of the activities of sympathetic nerves running within peripheral nerves and destined to skeletal muscles was investigated at rest in supine position (Macefield & Henderson, 2016). BOLD signal intensity showed that at complete rest in supine position, the active brain sites controlling the skeletal muscle sympathetic activity (MSNA) in peripheral nerves were CARS sites 13, 11, 8, 6, 3, and 1 (Figure 2). CARS sites 3, 6, and 8 are part of the limbic system while CARS site 11 is in the premotor area of the frontal lobe and CARS site 13 is in the precuneus cortex in the parietal lobe (Catani & Dell’Acqua, 2013). The limbic system controls emotion, memory and behaviors, while the premotor cortex is a supplementary motor cortex for learned and skilful motor activities and the precuneus cortex serves as a self‐consciousness and self‐processing area of the brain required in activities that involves self‐awareness (Catani & Dell’Acqua, 2013). The inhibition or restraint of MSNA at rest in supine position comes from only two CARS sites; 8 and 1, which means one CARS site in the limbic system and another in the brainstem and these two sites are active during both stimulation and inhibition of MSNA at rest in supine position. The pattern of central autonomic regulation shows bilateral and symmetrical activation of the precuneus cortex at CARS site 13, dorsolateral parts of the frontal lobe at CARS site 11 (the premotor area of the cortex), the posterior cingulate gyrus at CARS site 6, the ventromedial hypothalamus at CARS site 3 and the RVLM at CARS site 1 during bursts of MSNA at rest (Macefield & Henderson, 2016). Brain CARS site 8 comprises mid temporal lobe including uncus, entorhinal cortex and the parahippocampal gyrus. It also includes insula cortex, amygdala and hippocampus nuclei. There was asymmetric contralateral antagonism in central autonomic regulation indicated by activation of MSNA by the left mid‐insula cortex in CAR site 8 and inhibition of MSNA by the right ventral insular cortex also in CAR site 8 at rest in supine position (Macefield & Henderson, 2016). Brain CARS site 3 is in the hypothalamus and the ventromedial part that receives signals from CARS sites 8 and 9 (Figure 2) was active bilaterally as discussed before, but the dorsomedial part that receives signals from CARS sites 11 and 13 was only active on the left side during bursts of MSNA. It means CARS site 3 has a complex pattern of central autonomic regulation where one part of the site has bilateral and symmetrical activity but another segment of the same site shows unilateral activity during central autonomic regulation at rest in supine position. Brainstem CARS site 1 consists of rostral and caudal medulla oblongata as has been described before. There was bilateral and symmetrical inhibition and restrains of the bursts of MSNA by the cVLM and NTS. This is consistent with what has been observed and described in lower animals that the RVLM activates but the caudal medulla depresses or inhibits sympathetic activities especially the vasomotor tone and NTS is involved in this inhibition.

Takeaway points

1. Central activation of muscle sympathetic nerves at rest in supine position is symmetric and bilateral and comes from multiple sites in both upper and lower cortices of the cerebrum and from the brainstem. The premotor cortex of the frontal lobe and the precuneus cortex in the parietal lobe participate jointly in the symmetric activation from the upper cerebrums. The posterior cingulate cortex and the ventromedial hypothalamus, both of which are within the limbic system, together with the RVLM in the brainstem are the subcortical sites for symmetric and bilateral activation of MSNA at rest in supine position. These CARS sites are represented in Figure 2 as 13, 11, 6, 3, and 1.
2. There is, in addition, a unilateral activation site in the left dorsomedial hypothalamus within the limbic system. This is CARS site 4 in Figure 2.
3. There is asymmetric contralateral antagonism within the insular cortex in the limbic system where the left mid‐insular cortex participates in the activation but the right ventral insular cortex inhibits MSNA at rest in supine position. This is in CARS site 8 in Figure 2.
4. The main inhibition and restraint of MSNA at rest in supine position are from the brainstem in CARS site 1. This inhibition is bilateral and symmetrical and comes from the cVLM and NTS in the brainstem, both of which are situated in CARS site 1. This pattern of sympathetic regulation at the brainstem is identical to that described in lower animals including rodents.

###### Baseline regulation of cutaneous sympathetic activity in supine position

Baseline brain control of the activities of sympathetic nerves running within peripheral nerves and destined to the skin was investigated at rest in supine position (Macefield & Henderson, 2016). BOLD signal intensity showed that at complete rest in supine position, the active brain sites controlling the cutaneous sympathetic activity were CARS sites 13, 10, 9, 8, 6, and 4 (Figure 2). The inhibition or restraint of cutaneous sympathetic activity at rest in supine position came unilaterally from only one site and that is CARS site 9 on the left side of the brain. There were no involvements of CARS sites 3 and 1 in the regulation of cutaneous sympathetic activity at rest in supine position. The pattern of central autonomic regulation showed bilateral and symmetrical facilitation from CARS site 13 in the precuneus cortex and CARS site 6 in the mid cingulated cortex. There is asymmetric but bilateral facilitation in CARS site 8 arising from the right anterior insula cortex and the left posterior insula cortex. Unilateral facilitations are from the right anterior prefrontal cortex in CARS site 10 and the left ventro‐medial thalamus in CARS site 4. There is contralateral antagonism arising from CARS site 9 at rest in supine position in which facilitation of cutaneous sympathetic activity came from the right orbitofrontal cortex and inhibition came from the left orbitofrontal cortex.

Takeaway points

1. Central regulation of cutaneous sympathetic activity at rest is within the limbic system and is restricted to supra‐medullary sites with little or no involvement of CARS sites below the aqueduct of sylvius.
2. There is only one unilateral CARS site for restraining cutaneous sympathetic activity at rest involving the contralateral antagonism in CARS site 9 where the left orbitofrontal cortex inhibits and the right orbitofrontal cortex facilitates.
3. There is symmetric and bilateral facilitation of cutaneous sympathetic activity at rest coming from the precuneus cortex in CARS site 13 and lower down from the mid cingulate cortex in CARS site 6.
4. There is asymmetric but bilateral facilitation of cutaneous sympathetic activity from CARS site 8 coming from the right anterior insula and the left posterior insula cortices.
5. There are, in addition, unilateral facilitations of cutaneous sympathetic activity coming from right anterior prefrontal cortex in CARS site 10 and the left ventro‐medial thalamus in CARS site 4.

##### Central autonomic regulation of heart rate in response to mild and moderate passive lower body venous pooling with no change in blood pressure

The BOLD signal intensity showed that at complete rest in supine position the CARS sites responding to mild lower body venous pooling without change in blood pressure caused by lower body negative pressure (LBNP) of −15 mmHg, equivalent to a drop in central venous pressure (CVP) from 7 mmHg to 5 mmHg (Kimmerly, O’Leary, Menon, Gati, & Shoemaker, 2005) and moderate lower body negative pressure (LBNP) of −35 mmHg, equivalent to a drop in CVP from 7 mmHg to 3.7 mmHg with significant increases in both heart rate (up to 112%) and MSNA (up to 300%) but without significant increase in blood pressure (Kimmerly et al., 2005) are 4, 6, 7, 8, 9, 10, 11, 12, 13, and 14 (Figure 2).

###### Pattern of central autonomic regulation in response to lower body venous pooling

The heart rate has tonic, symmetric, and bilateral restraint within the limbic system coming from the anterior cingulate cortex in CARS site 6, anterior insular cortex in CARS site 8, the amygdala in CARS site 8 and the medial dorsal nucleus of thalamus in CARS site 4 during mild LBNP. There is an additional unilateral restraint coming from the right orbitofrontal cortex in CARS site 9 (Figure 2). These tonic restrains are withdrawn in a complex pattern in response to mild LBNP. There is appropriate and bilateral reduction of the restrains from the anterior cingulate and anterior insula cortices, but are associated with unilateral withdrawals of the restraints from the left dorsal medial nucleus of the thalamus, the left amygdala and the right orbitofrontal cortex. However, the specific autonomic drive of heart rate in response to mild lower body venous pooling is unilateral and comes from the right side of CARS sites 8, 11, 12, and 14 (Figure 2). These are the right posterior insular cortex in CARS site 8, the right premotor cortex of the frontal lobe in CARS site 11, the right post‐central gyrus in CARS site 12 and the cerebellum in CARS site 14. Mild lower body venous pooling also provokes simultaneous unilateral autonomic drives mainly from the right frontal lobe to the cardiovascular system. The right prefrontal cortex in the inferior frontal gyrus in CARS site 10, right middle frontal gyrus in CARS site 11, right fronto‐parietal gyrus in CARS site 12, the right precuneus cortex in CARS site 13, and the left cerebellum in CARS site 14 (Figure 2) are all involved in providing the autonomic drives to the cardiovascular system in response to mild lower body venous pooling leading to a nonsignificant increase in the heart rate, but a significant increase in MSNA up to 192% without change in blood pressure (Kimmerly et al., 2005). These unilateral autonomic cardiovascular drives are intensified during moderate lower body venous pooling. Other parts of the frontal lobe with asymmetric but bilateral activities are the dorsolateral prefrontal cortices involving the left Brodmann area 46 within CARS site 10 and the right Brodmann area 9 also still within CARS site 10 (Figure 2). The symmetric and bilateral autonomic restraint within the limbic system is also upgraded during response to moderate compared with mild lower body venous pooling. There is, in addition, a unilateral withdrawal of the autonomic restraint coming from the right periaqueductal gray matter in the midbrain in CARS site 7 in response to moderate lower body venous pooling leading to the significant increases in heart rate and MSNA described above, but no significant increase in mean arterial blood pressure.

Takeaway points

1. Chronotropic function of the heart in humans is facilitated predominantly from multiple sites on the right side of the brain by specific groups of neurons with organotopic spatial arrangements. Recruitment of similar sites from the left side of the brain is used to upgrade chronotropic facilitation when there is significant increase of cardiovascular stress above baseline.
2. Current fMRI evidence suggests that the chronotropic drive from the brain comes from organotopic neurons situated within the premotor area of the frontal lobe, which is the general area responsible for the execution of learned and skillful motor activities. These chronotropic organotopic neurons are in CARS site 11 (Figure 2) and they are activated in synchrony with somatosensory neurons in the right CARS site 12. The right posterior insula cortex in CARS site 8 and the left cerebellum in CARS site 14 (Figure 2) also provide chronotropic drive. It therefore means altogether CARS sites 8, 11, and 14 provide the major chronotropic drive synchronized with somatosensory activation in CARS site 12 in response to lower body venous pooling.
3. Chronotropic function of the heart in humans is restrained and modulated for appropriateness and magnitude within the limbic system, which is also responsible for memory, emotion, and behaviors among other functions. This restraint, which extends to the periaqueductal gray matter in the midbrain, has to be subdued or reduced substantially during significant increase of cardiovascular stress above baseline. It is known that the periaqueductal gray matter modulates responses to threatening stimuli.
4. Current fMRI evidence suggests that the left amygdala, left medial dorsal nucleus of the thalamus and the right anterior cingulate cortex have groups of neurons with organotopic spatial arrangements and these neurons specifically restrain and modulate chronotropic function of the heart. Recruitment of similar sites from the opposite side of the brain is used to remove or substantially reduce the chronotropic restraint and modulation when there is a significant increase of cardiovascular stress above baseline. This recruitment to reduce the extra capacity for restraint is extended to the right posterior–inferior insula cortex and the left periaqueductal gray matter. It therefore means the restraint and modulation of chronotropic function of the heart in response to moderate lower body venous pooling normally comes from CARS sites 4, 6, 7, and 8 (Figure 2).
5. General autonomic drive in response to lower body venous pooling comes from the frontal lobe in CARS sites 12, 11, and 10 (Figure 2) and is closely monitored by the right precuneus cortex in CARS site 13. The precuneus cortex is responsible for self‐consciousness and self‐processing. Although there is asymmetric and bilateral autonomic drive from the dorsolateral prefrontal cortex in CARS site 10 (Figure 2), the right premotor area in CARS site 11 is dominant in proving general autonomic drive, while the left side is recruited when there is significant increase of cardiovascular stress above baseline.
6. General autonomic restraint and modulation in response to lower body venous pooling comes from the limbic system in CARS sites 6 and 8 (Figure 2). This is bilateral and symmetric and the restraint is substantially reduced mainly in the anterior insula and anterior cingulate cortices when there is significant increase of cardiovascular stress above baseline. Extra capacity for reduction of general autonomic restraint and modulation in response to lower body venous pooling comes from the right orbitofrontal cortex in CARS site 9 (Figure 2).

#### 1.3.2 Lessons from positron‐emission tomography (PET) studies in human

Positron‐emission tomography (PET) is a nuclear functional imaging technique that is used to track metabolic processes in the body, usually in real‐time. The system detects pairs of gamma rays emitted indirectly by positron emitting radioligands like ^18^F in the form of fludeoxyglucose (FDG), an analogue of glucose. The concentrations of this tracer when imaged in the body will indicate tissue metabolic activity and corresponds to the regional glucose uptake at that moment. Other radioligands like ^15^O in the form of water (H_2_^15^O) is used to indicate changes in regional blood flow in organs or tissues in the body. Three‐dimensional images of the tracer concentration within the body are constructed from gamma rays scanners by computer analysis. This gamma rays image is superimposed onto three‐dimensional images of the organ or the whole body acquired by other methods of scanning like x‐ray computed tomography (CT), magnetic resonance imaging (MRI), functional magnetic resonance imaging (fMRI), and ultrasound.

##### Central autonomic regulation of chronotropic response to isometric contraction of skeletal muscles of the hand with significant change in heart rate

###### Chronotropic drive during isometric contraction of skeletal muscles

The active positive chronotropic organotopic neurons during isometric contraction of skeletal muscles are in CARS sites 14, 8, and 1 (Figure 2). Human subjects underwent H_2_^15^O PET scanning while performing isometric contraction of skeletal muscles using their right hand and the heart rate (HR) was monitored during scanning (Critchley, Corfield, Chandler, Mathias, & Dolan, 2000). This study showed that in order to achieve a significant increase in HR, there are significant increases in blood flow in two cerebellar peduncles; the right inferior and the left middle peduncles carrying proprioceptive and vestibular inputs and outputs. This is associated with significant increases in blood flow only in the right retrofacial area in the lower pons within CARS site 1 and the left cerebellum in CARS site 14 and also in the right insula cortex in CARS site 8 (Figure 2). This means although there are significant and bilateral increases of proprioceptive inputs into both left and right cerebella during unilateral isometric contraction of skeletal muscles in the right hand, only one cerebellum on the contralateral left side provides the heart with significant chronotropic drive, assisted by ipsilateral CARS sites in the right insula cortex and the brainstem tachycardia area also on the right side. It can be concluded that chronotropic drives come from the contralateral cerebellum in CARS site 14 on the left side and the ipsilateral insula cortex in CARS site 8 on the right side, and both act on the organotopic chronotropic neurons in the tachycardia area of the brainstem on the right side in CARS site 1 during isometric contraction of skeletal muscles in the right hand. Two of the supramedullary positive chronotropic CARS sites 14 and 8 are similar to what was observed above during lower body venous pooling.

###### Chronotropic restraint during isometric contraction of skeletal muscles

The active negative chronotropic organotopic neurons during isometric contraction of skeletal muscles are all within the limbic system in CARS sites 10, 9, 8, and 6 (Figure 2). The PET study discussed above shows significant and bilateral reduction of blood flow in the orbitofrontal cortices in CARS site 9. There are, in addition, significant reductions of blood flows in the left amygdala in CARS site 8, the right anterior and posterior cingulate cortex in CARS site 6 and the right middle frontal gyrus in the frontal eye fields in CARS site 10. This shows that the major chronotropic restraint applied to the heart during isometric contraction of skeletal muscles of the right hand comes from the left amygdala and the right anterior cingulate cortex in CARS sites 8 and 6, respectively. These two negative chronotropic CARS sites are similar to what was observed above during lower body venous pooling. The PET study also shows that the orbitofrontal cortex in CARS site 9, which is involved in the regulation of sympathetic functions in the skin as discussed above, is significantly and bilaterally subdued in order to achieve significant increase in chronotropic function of the heart during isometric contraction of skeletal muscles.

##### Central autonomic regulation of arterial blood pressure in response to isometric contraction of skeletal muscles of the hand with significant change in mean arterial blood pressure

###### Arterial pressure drive during isometric contraction of skeletal muscles

The arterial pressure drives come from CARS sites 14, 12, 10, 8, and 6 (Figure 2). Human subjects underwent H_2_^15^O PET scanning while performing isometric contraction of skeletal muscles using their right hand and the mean arterial pressure (MAP) was monitored during scanning (Critchley et al., 2000). This study shows that in order to achieve a significant increase in MAP, there is significant and bilateral increase in blood flow in both left and right cerebella in CARS site 14. There are, in addition, increases in blood flows in the left primary somatosensory cortex at Brodmann area 2 in CARS site 12, the right anterior prefrontal cortex in CARS site 10, the right anterior insula cortex in CARS site 8 and the right anterior cingulate cortex in CARS site 6. This means the two cerebella drive the increase in arterial blood pressure bilaterally with the assistance of the contralateral somatosensory cortex during unilateral isometric contraction of skeletal muscles on only one side of the body. There are additional drives from multiple sites within the limbic system on the ipsilateral side of the contracting muscles.

###### Restraints of the arterial pressure rise during isometric contraction of skeletal muscles

The restraints of arterial pressure increase during isometric contraction of muscles are from within the limbic system in CARS sites 9, 8, and 6 (Figure 2). Human subjects underwent H_2_^15^O PET scanning while performing isometric contraction of skeletal muscles using their right hand and the mean arterial pressure (MAP) was monitored during scanning (Critchley et al., 2000). This study shows bilateral reduction of blood flow in the orbitofrontal cortices in CARS site 9 associated with the rise in arterial pressure during isometric contraction of skeletal muscles. There are, in addition, unilateral and significantly reduced blood flows on the left side in the uncus in CARS site 8, the hippocampus in CARS site 8 and the posterior cingulate cortex in CARS site 6. On the right side there are reduced blood flows in the middle temporal gyrus in CARS site 8 and the parahippocampal gyrus also in CARS site 8. This again shows that the orbitofrontal cortex in CARS site 9, which is also involved in the regulation of sympathetic functions in the skin as discussed before, is significantly and bilaterally subdued in order to achieve significant increase in arterial blood pressure during isometric contraction of skeletal muscles. The PET study also shows that there is asymmetric contralateral antagonism within CARS site 6 where activity in the right anterior cingulate cortex increases with the rise in arterial blood pressure and the activity in the left posterior cingulate cortex is subdued or reduced at the same time. This means the limbic system bilaterally restraints the rise in arterial blood pressure during unilateral isometric contraction of skeletal muscles using mainly organotopic neurons in multiple areas situated within CARS sites 8 and 9.

Takeaway points

1. There are concurrent and significant increases in both heart rate and arterial blood pressure during isometric contraction of skeletal muscles even when there is no effort in performing the task, but the magnitudes of changes increase with efforts.
2. Propioceptive activation of the contralateral cerebellum in CARS site 14 provides positive chronotropic drive to the ipsilateral organotopic chronotropic neurons in the tachycardia area of the brainstem in CARS site 1.
3. Somatosensory activation of the contralateral cerebral cortex in CARS site 12 in combination with propioceptive activation of both left and right cerebella in CARS site 14 altogether drive the arterial blood pressure rise during isometric contraction of skeletal muscles.
4. The limbic system restrains and modulates the magnitudes of both the chronotropic effect and the arterial blood pressure rise during isometric contraction of skeletal muscles through ipsilateral facilitations combined with bilateral restrains by organotopic neurons situated in different areas in CARS sites 10, 8, and 6.
5. The orbitofrontal cortices in CARS site 9, which are also known to regulate autonomic functions in the skin, are bilaterally and significantly subdued during isometric contraction of skeletal muscles to allow concurrent rise of both heart rate and blood pressure. Since the gradient of concurrent changes of arterial blood pressure and heart rate is a measure of baroreflex gain, it is evident that a major role of CARS site 9 is to set and maintain the central baroreflex gain during contraction of skeletal muscles.

### 1.4 Physiology of orthostatic tachycardia

#### 1.4.1 A model of cardiovascular regulation to facilitate our understanding of the physiology of orthostatic tachycardia

**FIGURE 3:**
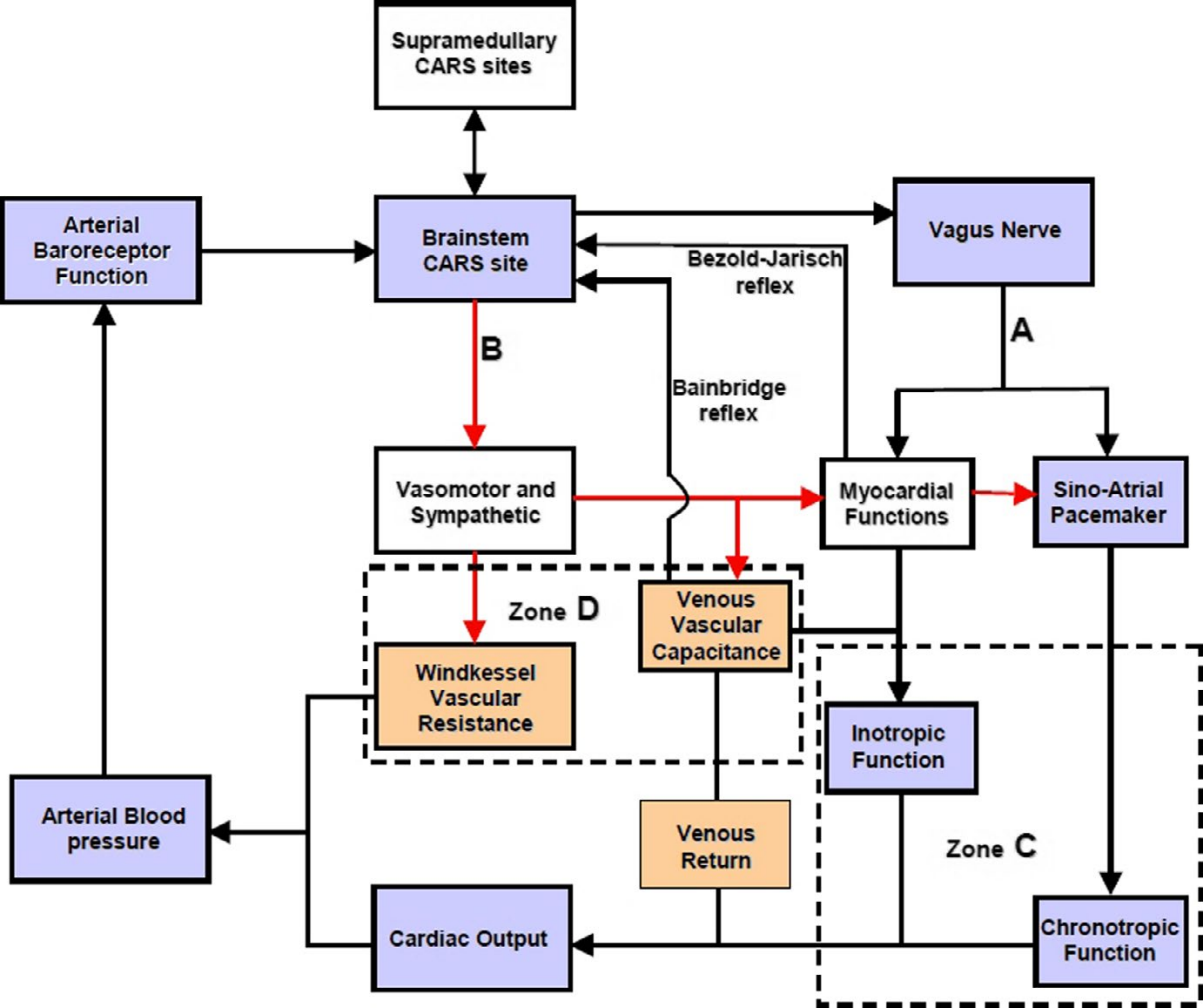
Proposed model of beat‐to‐beat cardiovascular regulation. The reflex negative feedback control is executed within the brainstem central autonomic regulation site (CARS) using arterial baroreceptor afferent and two efferent pathways A and B. The supramedullary CARS sites adjust the magnitude of the reflex regularly. The descending pathway A = vagus nerve with two tonic parasympathetic pathways that regulate inotropic and chronotropic functions of the heart in Zone C. B = the common sympathetic and vasomotor pathway descending in the spinal cord with the vasomotor efferent that regulates windkessel vascular resistance and venous vascular capacitance in Zone D. The venous vascular capacitance has afferent input to the brainstem CARS site coming from what are known as volume receptors. These mediate the Bainbridge reflex. The myocardium also has afferent input to the brainstem CARS site and this mediate the Bezold‐Jarisch reflex. The resultant venous return emerging from capacitive vessels is transformed into cardiac output by the combined chronotropic and inotropic efforts of the heart. The combined effects of cardiac output into windkessel vessels and controlled drainage of blood away from the windkessel vessels by windkessel vascular resistance generate arterial blood pressure (see text for full description of the model). Medical technology is available for continuous and real‐time measurements of the cardiovascular functions in the light blue boxes. Laboratory standardized surrogate measures of cardiovascular functions in the light coral boxes are also available

We propose here a model of beat‐to‐beat cardiovascular regulation based on the current knowledge of functional anatomy discussed in detail in the previous section (Figure 3). This model will be used to explain the regulatory changes during a laboratory‐controlled and progressive increase in orthostatic stress leading up to failure of cardiovascular compensation and the beginning of pre‐syncope with subsequent failure to maintain a physiologically viable arterial blood pressure during orthostasis. All major cardiovascular parameters will be monitored and if possible quantified during the progressive orthostatic stress, particularly highlighting the role of the heart rate in every step of cardiovascular compensation and during decompensation and subsequent failure to maintain a viable arterial blood pressure. This model will be used to explain the physiology behind excessive tachycardia during orthostasis in a normal human population.

#### 1.4.2 Proposed model of beat‐to‐beat cardiovascular regulation

##### The role of supramedullary CARS sites in this model

In the proposed model (Figure 3), all the supramedullary CARS sites starting from 3 to 14 (Figure 2) are considered together as processing sites with a common task to deliver to the brainstem autonomic drives that is appropriate for execution in a given physiological state of the body at that moment. The detailed discussion of functional anatomy in the previous section can be condensed to reflect that the supramedullary CARS sites use sensory inputs from the primary somatosensory areas of the cortex to process within the precuneus cortex, the self‐awareness and self‐consciousness according to the prevailing physiological state. Crude autonomic drives in response to the prevailing physiological states are activated from the premotor area of the frontal cortex assisted by the cerebellum. These crude autonomic drives are further modulated and refined by the limbic system through restrains and or additional stimulations and delivered for execution to appropriate organotopic neurons in CARS site 1 in the brainstem (Figure 2). There is bidirectional communication between the brainstem and the supramedullary CARS sites (Figure 3) to facilitate feedback and further adjustments of the autonomic drives to match the continuously changing physiological state of the body.

##### The role of the brainstem in this model

The brainstem CARS site 1 is the major central beat‐to‐beat cardiovascular regulatory site in this model receiving the necessary beat‐to‐beat arterial baroreceptor input required for immediate cardiovascular adjustments (Figure 3). This means the regulatory target in this model is the arterial blood pressure and the effective perfusion pressure in a cardiac cycle is the mean arterial pressure (MAP). Therefore, the model proposes that a target MAP appropriate for the prevailing physiological state is defended by continuous beat‐to‐beat adjustments of the following cardiovascular functions: two cardiac functions, the inotropic and chronotropic contractions of the heart (Zone C in Figure 3) are adjusted beat‐by‐beat concurrently with two vascular functions; the total windkessel vascular resistance to regulate arterial drainage and the total venous vascular capacitance to regulate the volume of venous blood returned back to the heart commonly referred to as venous return (Zone D in Figure 3). The regulatory commands for beat‐to‐beat cardiovascular adjustments are delivered through two descending pathways originating from CARS site 1 in the brainstem (Figure 2). These are the common parasympathetic pathway in the vagus nerve (A in Figure 3) and the common sympathetic pathway in the spinal cord (B in Figure 3). The origins of these common autonomic pathways are discussed in detail in the previous section. Due to the organotopic arrangements of neurons in CARS sites discussed before, the regulation of chronotropic function of the heart is independent to that of its inotropic function and likewise, the regulation of windkessel vascular resistance to control beat‐to‐beat arterial drainage of blood is independent to the regulation of venous vascular capacitance to control beat‐to‐beat blood volume returned to the heart, or venous return.

##### The role of tachycardia in the beat‐to‐beat model of cardiovascular regulation

In an example discussed in the previous section above, moderate lower body venous pooling induced by external negative lower body pressure reduced the central venous pressure to 50% of the initial normal resting value. Muscle sympathetic nerve activity (MSNA) increase to 300% of the initial resting value and the heart rate increased by 19% in order to successfully defend the resting supine MAP, which did not change significantly during the external negative lower body pressure challenge. The reduced central venous pressure suggests that a reduction of the venous blood volume returned to the heart is the main cardiovascular stress in this example. The best way to have ascertained this would be to measure the cardiac output during this study, but it was not done. However, reduced venous return could have been achieved by mechanical manipulation of the venous vascular capacitance using negative pressure to suck, distend and enlarge the veins due to the much higher compliance of venous walls compared to arterial walls. Venous wall has about thirty times more compliance than arterial wall (Gelman, 2008). Increased venous vascular capacitance caused by the mechanically distended veins causes venous pooling and reduces venous return. The increased MSNA represents an upgrade of the total windkessel vascular resistance and the increased heart rate represents an upgrade of the chronotropic function of the heart in response to this cardiovascular stress. Inotropic function of the heart is one other independent cardiovascular parameter proposed in our model (Figure 3) but it was not monitored in this example. It is evident in this example that tachycardia is part of a compensatory mechanism used during cardiovascular stress involving reduced venous return.

#### 1.4.3 Two forces generate orthostatic cardiovascular stress

##### Gravity

Gravity creates vascular hydrostatic pressure, which is proportional to the density of blood and the vertical height of the column of blood inside the blood vessel. Since gravitational force during orthostasis is downwards toward the center of the earth, the hydrostatic pressure in all the venous vascular system below the horizontal level of the heart is in opposition to venous return to the heart. Venous hydrostatic pressure facilitates pooling of blood within the veins due to the high compliance of venous vascular walls as discussed before. In other words, gravity increases the total venous vascular capacitance shown in Figure 3 in proportion to the vertical heights of the respective venous vascular columns. The longer the vein, the greater is the potential gravity‐induced increase in venous vascular capacitance in the upright position. Increased venous vascular capacitance causes cardiovascular stress by reducing venous return thereby activating compensatory measures to counteract the stress in order to defend a target MAP appropriate for the prevailing physiological state. The total gravity‐induced increase in venous vascular capacitance is larger in the upright position compared to the supine position. In an example of acute loss of gravity during a parabolic flight in an aeroplane, a brief loss of gravity‐induced venous vascular capacitance during zero gravity lasting only 20 s caused a 23% increase in cardiac output (Petersen, Damgaard, Petersen, & Norsk, 2011) equivalent to 23% increase in venous return in subjects sitting upright. Loss of gravity‐induced venous vascular capacitance for the same duration increased cardiac output by only 11% in subjects lying in supine position (Petersen et al., 2011) equivalent to 11% increase in venous return, which is about 50% of that observed in the sitting position. This example shows that gravity increases venous vascular capacitance to reduce venous return and cardiac output (Figure 3) and this gravity‐induced cardiovascular stress is more than twice as large when sitting in an upright position compared with the cardiovascular stress when lying in supine position. Cardiovascular compensatory mechanisms during reduced venous return involve tachycardia as discussed before in the example of venous pooling during lower body negative pressure. It therefore means gravity‐induced tachycardia increases during the change in body position from supine to upright. There are multiple organotopic neurons in supramedullary CARS sites involved in this positive chronotropic change, most of these CARS sites are on the right‐hand side of the brain as discussed in the previous section. Gravity‐induced reduction of venous return activates somatosensory neurons in the right CARS site 12 (Figure 2) concurrently with organotopic chronotropic neurons in the premotor area of the right frontal lobe in CARS site 11 (Figure 2). The contralateral left cerebellum in CARS site 14 is also activated to provide positive chronotropic drive together with the modulations from the limbic system, which consist largely of the concurrent withdrawal of baseline restraint from the amydala and facilitation from the right posterior insular cortex, altogether constitute supramedullary chronotropic command to the organotopic cardioaccelerator neurons in the brainstem CARS site (Figure 3). Gravity‐induced chronotropic commands from the brainstem targeting cardiac functions (Zone C, Figure 3) are delivered through two descending autonomic common pathways; one in the vagus nerve (pathway A, Figure 3) and the other in the spinal cord (pathway B, Figure 3). Detailed functional anatomy of autonomic response to reduced venous return is discussed in the previous section. It includes the significant increase in MSNA interpreted here to represent the adjustment of windkessel vascular resistance in response to an increase in venous vascular capacitance (Zone D, Figure 3).

Takeaway points

1. Gravity increases the total venous vascular capacitance by creating hydrostatic pressure that distends the high compliance venous walls leading to pooling effect
2. Gravity‐induced increase in venous capacitance holds back more than 10% of the cardiac output in the supine position in humans. This is however more than doubled in the upright position, when gravity‐induced increase in venous capacitance holds back more than 20% of cardiac output even in an upright sitting position
3. Tachycardia is part of the natural compensatory mechanism in response to reduced venous return and is used to defend the appropriate target arterial blood pressure required for the prevailing physiological state

##### Skeletal muscles contraction

The process of orientation to an upright stance and the balance to maintain the upright stance against gravity during orthostasis involves contraction of skeletal muscles. These muscles are sometimes described as antigravity (Sherrington, 1910) or postural muscles. Postural muscles are enriched with slow‐twitch or tonic muscle fibers for isometric contraction (Ivanenko & Gurfinkel, 2018) and they include the large muscles in the neck, trunk and lower limbs. All muscle contractions, particularly isometric contractions, induce cardiovascular stress by creating sudden and large metabolic demand. Skeletal muscle metabolic demands significantly stimulate the autonomic nervous system by concurrent removal or reduction of baseline restraints while actively exciting specific organotopic neurons in the brain including the brainstem as discussed before. Somatosensory inputs to the supramedullary CARS sites (Figure 3) arising from the contracting muscles and other organs include propioceptive and vestibular inputs. These are the main drives of the autonomic nervous system during isometric contraction of skeletal muscles during orthostasis (see Functional Anatomy above). However, the organotopic cardioaccelerator neurons in CARS site 1 in the brainstem respond more closely to the propioceptive and vestibular inputs during isometric contraction of skeletal muscles and the responses are modulated by the limbic system as discussed before. The overall effects of the concurrent withdrawal of baseline autonomic restraint combined with stimulation of specific organotopic neurons in supramedullary CARS sites are to target and adjust cardiac functions (Zone C, Figure 3) and vascular functions (Zone D, Figure 3) to meet the new metabolic demand. This autonomic command is transmitted to the organotopic neurons in the brainstem CARS site and executed through two descending autonomic common pathways (A and B, Figure 3) leading to simultaneous and significant increases in both arterial blood pressure and tachycardia. The magnitude of autonomic excitation during isometric contraction of skeletal muscles is related to the contracting muscle mass (Seals, 1989) and the gradient of concurrent increases in heart rate and arterial blood requires significant reduction of the restraint from CARS site 9. The two simultaneous cardiovascular effects of isometric contraction of skeletal muscles are significant even when the muscle contractions are effortless as discussed above in Functional Anatomy. It is therefore evident that in order to achieve a proper resting baseline autonomic state, all skeletal muscles except the respiratory muscles, should be fully relaxed and the subject should be in a supine position for minimal gravitational cardiovascular stress.

Takeaway points

1. Antigravity skeletal muscles create cardiovascular stress during orthostasis by performing isometric contractions. This creates sudden and sustained increase in metabolic demand and a new physiological state that requires increased arterial blood pressure is set up
2. Isometric contraction of skeletal muscles causes concurrent increases in arterial blood pressure and tachycardia caused by simultaneous withdrawal of baseline cardiovascular restraints and excitation of specific organotopic neurons in CARS sites in the brain, brainstem and the cerebellum
3. The organotopic cardioaccelerator neurons in the brainstem CARS site respond more closely to the proprioceptive and vestibular inputs during isometric contractions of skeletal muscles
4. The magnitude of autonomic excitation during isometric contraction is related to the contracting skeletal muscle mass, but CARS sites within the limbic system, particularly CARS site 9, restrain the extents of concurrent increases in heart rate and arterial blood pressure during muscle contraction.

#### 1.4.4 Laboratory‐controlled and progressive orthostatic stress

There are four phases of orthostasis revealed during a study of cardiovascular control during the period leading to pre‐syncope in patients with posturally related syncopal attacks (Julu, Cooper, Hansen, & Hainsworth, 2003). Briefly, men aged 30–55 years (mean ± *SEM*, 35 ± 8 years) and women aged 35–50 years (40 ± 5 years) volunteered in this study. All volunteers did not have any known cardiovascular or neurological disorders and were not taking any medications. Important beat‐to‐beat cardiovascular parameters including heart rate (HR) representing chronotropic function of the heart, systolic (SBP), diastolic (DBP), the mean arterial blood pressures (MAP), and forearm vascular resistance (FVR), all representing windkessel vascular functions (Figure 3) were monitored continuously. The following two indices of brainstem cardiovascular regulations: cardiac vagal tone (CVT) representing the active beat‐to‐beat central restraint of chronotropic function of the heart (Zone C, Figure 3) and cardiac sensitivity to baroreflex (CSB) representing the beat‐to‐beat negative feedback baroreflex gain the brainstem is using to regulate sympathetic vascular functions (Zone D, Figure 3) were also monitored and quantified continuously. We shall refer to CSB as beat‐to‐beat central sympathetic restraint because it is set up and frequently updated within the NTS of cVLM in the brainstem as previously discussed in Functional Anatomy. All the volunteers had normal supine baseline cardiovascular parameters as follows: MAP (means ± *SEM*) 84.9 ± 3.2 mmHg; HR, 63.9 ± 3.2 beats/min; CVT, 10.8 ± 2.6 LVS units (LVS, Linear Vagal Scale (Julu, 1992)) and CSB, 8.2 ± 1.6 ms.mmHg^−1^. A progressive orthostatic stress was achieved using head‐up tilt (HUT) at 60 degrees, followed by a combination of HUT and lower body suction (LBNP) to achieve pre‐syncope. We defined pre‐syncope as a fall in SBP to below 80 mmHg accompanied by symptoms. All volunteers reached this level of cardiovascular decompensation.

##### The phases of orthostasis

###### Early Part of Orthostasis in Phase 1

Phase 1 of orthostasis has early and late parts. The early part of phase 1 consists of several events that provoke readjustments of cardiovascular regulations. These events can be illustrated much better in the orthostasis during active standing more than during passive tilt. We shall therefore use the sequence of events illustrated in Figure 1 during active standing to illustrate the readjustments of cardiovascular regulation provoked by various events during early orthostasis starting with the command to achieve an upright stance and follow through to the full cardiovascular compensation during the upright stance (from a to f, Figure 1). The emphasis of this discussion will be on the regulation of heart rate during orthostasis. The command can be internal in the form of a decision to stand up or external when a subject is asked to stand up from a sitting position like in this example (a, Figure 1). The command causes immediate reduction of the beat‐to‐beat baroreflex negative feedback used for central sympathetic restraint and quantified as CSB (a, Figure 1), but the central restraint of the chronotropic function of the heart quantified as CVT is reduced a little later after a time lag following the orthostatic command (b, Figure 1). The ramp increase in heart rate during the early Phase 1 of orthostasis starts simultaneously with CVT withdrawal (b, Figure 1) earlier than any voluntary movement of the body at the beginning of orthostasis (see body movement artefacts in the blood pressure trace, Figure 1) and it ends with the restoration of CVT back to a level similar to that at the beginning of CVT withdrawal (d, Figure 1). The rebound bradycardia of orthostasis (starting from d to e, Figure 1) coincides with additional increases of both CSB and CVT exceeding the levels before the orthostatic command. Full cardiovascular compensation during which the heart rate is stable during orthostasis is achieved within 128 s, just over 2 min of actively standing upright in this example (starting from c to f, Figure 1).

Takeaway points

1. Cardiovascular arousal is indicated by the beginning of withdrawal of baroreflex central sympathetic restraint measured here as CSB. It starts at the same time with the command to execute orthostasis and precedes all other cardiovascular events during the orthostatic process
2. The start of the ramp increase in heart rate during early orthostasis coincides with the beginning of withdrawal of central chronotropic restraint measured here as CVT. The withdrawal of CVT and increase in heart rate precedes any body movement during the initiation of orthostasis
3. Changes in heart rate in early orthostasis have three stages; a ramp tachycardia followed by rebound bradycardia followed by recovery to stable but increased heart rate compared with the supine baseline level. The increase is about 17% above baseline level, with an average increase of 12 beats/min and a range of 5 to 25 beats/min above baseline level
4. The magnitudes of heart rate changes in early orthostasis have been standardized as a 30:15 ratio test and adopted for use in clinical examinations of autonomic functions
5. Full cardiovascular compensation in orthostasis is achieved within 3 min of assuming an upright stance. The consensus statement on the definition of orthostatic hypotension recommend assessment of postural hypotension at three minutes in upright or tilt position

###### The late part of orthostasis in phase 1

The late part of orthostasis Phase 1 starts at full cardiovascular compensation (starting from f, Figure 1). However, we shall use the example of laboratory‐controlled orthostatic stress during 60 degrees tilt to illustrate full cardiovascular compensation during orthostasis. Most healthy people can maintain full cardiovascular compensation during orthostasis at 60 degrees tilt for more than 20 min. In this example of laboratory‐controlled orthostatic stress, nearly 65% of the volunteers maintained full cardiovascular compensation for more than 20 min at 60 degrees tilt until LBNP was applied to achieve laboratory‐controlled cardiovascular decompensation. The final adjustments of cardiovascular regulation to achieve full cardiovascular compensation include increase of windkessel vascular resistance (Zone D, Figure 3) to 170% of supine baseline level and an upgrade of the chronotropic function of the heart (Zone C, Figure 3) to 17% above the supine baseline level leading to increase in heart rate by an average of 10 beats/min among the volunteers. The increased windkessel vascular resistance during orthostasis significantly increased the diastolic and the mean arterial blood pressure, but it did not significantly affect the systolic blood pressure. The adjustments of central cardiovascular regulations required for achieving full compensation at a slight but significantly raised MAP (6% above supine level) during orthostasis involve reduction of CVT to 32% and CSB to 33% of the supine baseline levels. It means all the parasympathetic restraints used for cardiovascular control at 60 degrees tilt are reduced to only one‐third of the baseline level in supine position to achieve full cardiovascular compensation.

Takeaway points

1. The majority of healthy people can maintain full cardiovascular compensation during orthostasis for more than 20 min at 60 degrees tilt
2. The international consensus statement on the definition of postural tachycardia syndrome (POTS) recommend a sustained heart rate increment of more than 30 beats/min within 10 min of orthostasis, but without orthostatic hypotension as a basis of the diagnosis of the disorder (Freeman et al., 2011). Conversely, this is equivalent to natural and physiological Phase 2 orthostasis observed in all volunteers in a laboratory‐controlled progressive orthostatic stress to be described later in this review
3. Full cardiovascular compensation during orthostasis is achieved by the application of the maximum windkessel vascular resistance leaving no reserve for any further and additional cardiovascular stress beyond the initial fully compensated status. This is equivalent to an all or none regulatory strategy in the use of windkessel vascular resistance during orthostasis
4. Full cardiovascular compensation during orthostasis is achieved by the reductions of the two central parasympathetic restraints of cardiovascular controls, CSB and CVT, to one‐third of their baseline supine values. Where CSB represents the baroreflex central sympathetic restraint and CVT represents the central chronotropic restraint of the heart. However, reserve capacity for cardiovascular regulation is still available for use should there be further and additional cardiovascular stress beyond the initial fully compensated status during Phase 1 orthostasis

###### Orthostasis phase 2

**FIGURE 4:**
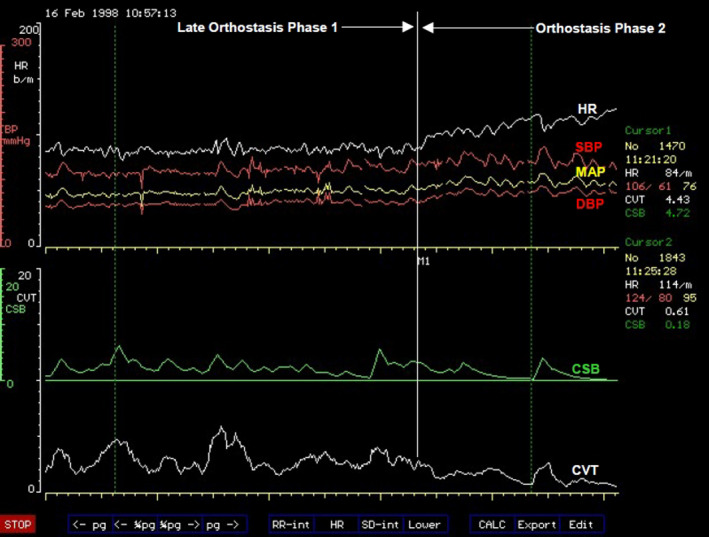
A computer screen capture from the NeuroScope displaying the following beat‐to‐beat traces of cardiovascular parameters at the transition of orthostasis Phase 1 to Phase 2. Heart rate (HR) expressed in beats.min^−1^; systolic (SBP), diastolic (DBP) and mean (MAP) arterial blood pressures measured in mmHg; cardiac sensitivity to baroreflex (CSB) measured in ms.mmHg^−1^ and cardiac vagal tone (CVT) measure in units of a linear vagal scale (LVS) (see text for descriptions and methods used to measure these cardiovascular parameters). Note the sudden onset of sustained and excessive tachycardia coinciding with further reduction of the already very low levels of CSB and CVT at the beginning of orthostasis Phase 2

Abrupt and progressive increase in heart rate marks the beginning of Orthostasis Phase 2 (Figure 4). It follows a period of stable heart rate in the late part of Phase 1 of orthostasis with full cardiovascular compensation. This is an early sign of severe orthostatic stress and can be largely asymptomatic but subjects may experience palpitations in the later part of this phase of orthostasis. The amplitude of blood pressure variation increases during this phase of orthostasis (Figure 4) and there is also a degree of overlap between this phase and the next Phase 3 of orthostasis. There is a sudden decrease in pulse pressure (measured in this example as the difference between beat‐to‐beat systolic and diastolic pressures) toward the end of Phase 2, which could be seen in all the volunteers in this example. The average duration of Phase 2 orthostasis is 4 min. The heart rate will either continue to increase up to the time when both blood pressure and heart rate becomes unstable and starts oscillating, or would reach a peak where it remains relatively stable for the remainder of this orthostatic phase. The heart rate reached an average 104 beats/min at the peak among volunteers in this phase of orthostasis in this laboratory‐controlled progressive orthostatic stress. This is a significant additional 39% increase in the chronotropic function of the heart (Zone C, Figure 3) above the level at the late part of Phase 1 of orthostasis described above. The central restraint of chronotropic function of the heart, represented by CVT in this example, was further reduced by a comparable proportion of 38% of the level at full cardiovascular compensation in the late part of Phase 1 orthostasis. There is a markedly significant rise of heart rate to a level that is 63% above the supine baseline level, which is an increase of 40 beats/min. With the CVT level of 2.2 ± 0.5 LVS units, it is a significant reduction by 80% during this phase of orthostasis compared with the baseline level in supine position. Interestingly, there is no further increase in forearm vascular resistance during this phase of orthostasis. There is instead a 16% reduction of forearm vascular resistance compared with the level during full cardiovascular compensation in the late part of Phase 1 from 170% to 144% of supine baseline level toward the end of Phase 2 orthostasis. This means the windkessel vascular resistance (Zone D, Figure 3) is downgraded by up to 16% in Phase 2 orthostasis compared to the level required for full cardiovascular compensation without excessive tachycardia during orthostasis. This is in spite of a further significant 56% additional reduction of baroreflex negative feedback sympathetic restraint, represented here by CSB, compared with the level at full cardiovascular compensation during the late part of Phase 1 orthostasis. With the CSB level of only 1.2 ± 0.4 ms.mmHg^−1^ there is in total 85% reduction of baroreflex central sympathetic restraint during this phase of orthostasis compared with the baseline level in supine position. This means all the parasympathetic restraints used for cardiovascular control during the severe orthostatic stress in Phase 2 orthostasis are reduced to less than 20% of the baseline level in supine position. Systolic and mean arterial blood pressures decreased by just 8% but with statistical significance compared with levels at full cardiovascular compensation, but there is no significant change in the diastolic pressure during this phase of orthostasis. Therefore, it is evident in this example that Phase 2 is a tachycardia phase of orthostatic cardiovascular compensation during which windkessel vascular resistance is either at the beginning of failure or it is being degraded due to metabolic demands. We can speculate that probably metabolic demands meant there is need for windkessel vascular resistance to be reduced in order to increase arterial drainage into tissues because the severe orthostatic stress applied during this phase would have reduced cardiac output (Zone D, Figure 3). As discussed above in Functional Anatomy, both gravity and LBNP do reduce cardiac output. Reduced cardiac output will only be confirmed as a cause for degrading the windkessel vascular resistance in this phase of orthostasis when beat‐to‐beat cardiac output is measured simultaneously with the other cardiovascular parameters (Zones C and D, Figure 3). This was not done during the laboratory‐controlled orthostatic stress in this example.

This observation means the chronotropic function of the heart (Zone C, Figure 3) takes the main role of cardiovascular compensation during orthostasis when the windkessel vascular resistance (Zone D, Figure 3) is failing or is degraded for metabolic reasons. This will require bilateral activations of chronotropic drive of the heart from the premotor areas of the frontal lobes in CARS site 11 (Figure 2) assisted by the right posterior insular cortex and the left cerebellum together with bilateral and near total withdrawal of chronotropic restraints from the amygdala, medial dorsal nucleus of the thalamus and the periaqueductal grey matters in the brainstem because the extent of this orthostatic stress is equivalent to moderate to severe loss of venous return as previously discussed in Functional Anatomy. The observed effects in this laboratory‐controlled orthostatic stress is the near total removal of the central parasympathetic restraints of cardiovascular regulations during which both CVT and CSB are reduced to their lowest levels that can support physiological functions of the cardiovascular system. This is associated with tachycardia at the highest level during the whole period of the controlled orthostatic stress. The clinical implication of this observation is that the administration of β‐adrenergic blockers or other centrally acting drugs like clonidine for treating the tachycardia of orthostasis must be done with caution in case this major and possibly the only compensatory mechanism available to the patient is disabled by drugs thereby severely affecting orthostatic tolerance.

Takeaway points

1. Degradation of the windkessel vascular resistance (Zone D, Figure 3) by an average of 16% below its maximum is a significant event in Phase 2 orthostasis. It triggers sudden and sustained tachycardia of the magnitude described in POTS
2. This is a tachycardia phase of orthostasis where the chronotropic function of the heart (Zone C, Figure 3) takes over as the main compensatory mechanism during a severe orthostatic stress accompanied by a significant 8% drop of MAP below the level in the initial full cardiovascular compensation status
3. The central parasympathetic restraints of cardiovascular functions, CSB and CVT, are reduced to their lowest levels that can sustain stable blood circulation in Phase 2 orthostasis. The reserve for functional parasympathetic withdrawal in cardiovascular control is exhausted in Phase 2 orthostasis
4. Supramedullary, CARS sites that restrain the chronotropic function of the heart (Zone C, Figure 3) in response to severe cardiovascular stress involving reduced venous return should be at their lowest activities during Phase 2 orthostasis (see Functional Anatomy for a detailed discussion). The main bilateral sites are the amygdala in CARS site 8 and the medial dorsal nucleus of the thalamus in CARS site 4 (Figure 2). Both are within the limbic system and are assisted by the right periaqueductal grey matter in the midbrain in CARS site 7. There is no evidence of inappropriate activities in these CARS sites during Phase 2 orthostasis because the magnitudes of both CSB and CVT stayed at their lowest as expected
5. The supramedullary CARS sites that actively increase the windkessel vascular resistance (Zone D, Figure 3) in response to severe cardiovascular stress involving reduced venous return have a reduced effectiveness in Phase 2 orthostasis below the level required for full cardiovascular compensation. The main bilateral sites are the premotor areas of the frontal lobes in CARS site 11 restrained and modulated by the anterior insula cortices within the limbic system in CARS site 8 (Figure 2). The activities of all these CARS sites are monitored closely by the precuneus cortex that normally processes self‐consciousness (see Functional Anatomy)

###### Orthostasis phase 3

**FIGURE 5:**
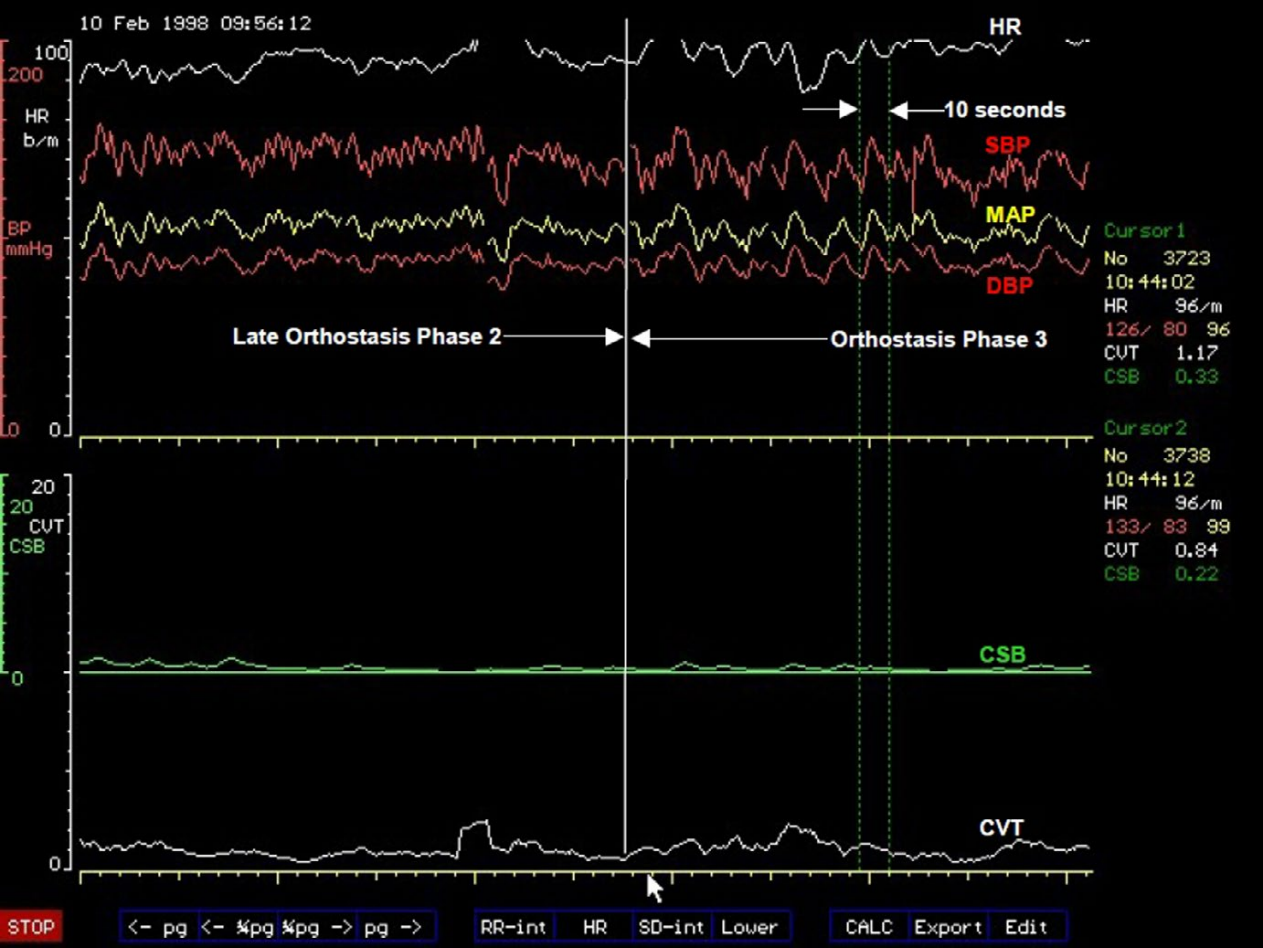
A computer screen capture from the NeuroScope displaying the following beat‐to‐beat traces of cardiovascular parameters at the transition of orthostasis Phase 2 to Phase 3. Heart rate (HR) expressed in beats.min^−1^; systolic (SBP), diastolic (DBP), and mean arterial blood pressures (MAP) measured in mmHg; cardiac sensitivity to baroreflex (CSB) measured in ms.mmHg^−1^ and cardiac vagal tone (CVT) measure in units of a linear vagal scale (LVS) (see text for descriptions and methods used to measure these cardiovascular parameters). Note the clearly demarcated border of increased amplitude of oscillations of blood pressure and heart rate at the beginning of orthostasis Phase 3 without any further changes in the already very low levels of CSB and CVT. The period of blood pressure oscillation is 10 s (frequency of 0.1 Hz)

This phase is symptomatic presenting with very early evidence of pre‐syncope like dizziness and is characterized by exaggerated oscillations of both arterial blood pressures and heart rate (Figure 5). Phase 3 tends to overlap with Phase 2 orthostasis. The average amplitude of the oscillations of systolic blood pressure is 27 mmHg with a period of oscillations of about 10 s (a frequency of 0.1 Hz). This is known as Traube–Hering–Mayer's waves (Julu et al., 1997; Sato, 1951). This is the mammalian cardiovascular natural resonance frequency caused by the feedback controls in the cardiovascular regulatory circuit (DeBoer, Karemaker, & Strackee, 1987). Traube–Hering–Mayer's waves can be recorded in mammals of various sizes under various conditions, for example, in humans (Delamont, Julu, & Jamal, 1999; Julu et al., 1997 ), in dogs (Little & Julu, 1995) or even in mice (Bumstead, Bauer, Wright, & Culver, 2017). The average values of systolic blood pressure during these oscillations actually do increase above the values observed during Phase 2 orthostasis. Heart rate is significantly less than it is during Phase 2 orthostasis with an average of 98 beats/min, It is 6% lower than its average peak in Phase 2 orthostasis but it is still significantly higher than the heart rate at baseline in supine position (Julu et al., 2003). The average duration of Phase 3 orthostasis is 2 min. Central cardiovascular regulation during Phase 3 orthostasis is not very different from that during Phase 2. The average value of CVT during this phase is 2.1 LVS units and average value of CSB is 1.3 ms.mmHg^−1^, not significantly different from that during Phase 2 orthostasis. It is therefore evident that the chronotropic function of the heart (Zone C, Figure 3), which has a major compensatory role in Phase 2, is beginning to be degraded or to fail in Phase 3 orthostasis. We can speculate here that it is the sympathetic chronotropic drives from the premotor areas of the frontal lobes assisted by the posterior insula cortex and the cerebellum that are selectively reduced in Phase 3 compared with the levels in Phase 2 orthostasis without any alteration of the levels of the limbic and midbrain parasympathetic restraints. This is because the levels of both CVT and CSB are kept at their lowest in this orthostatic phase, comparable to the levels in Phase 2 orthostasis. This means cardiovascular functions in both Zones C and D (Figure 3) are beginning to be either degraded or to fail during Phase 3 orthostasis. Both CVT and CSB, the two central parasympathetic restraints of cardiovascular regulations are kept at their lowest levels in response to this severe orthostatic stress in Phase 3 Orthostasis (Figure 5). The large amplitude of blood pressure oscillation suggests that there is insufficient dumping effect in the feedback control circuit of blood circulation. It means the levels of both CSB and CVT are too low to sustain stable blood circulation during the combined sub‐optimal chronotropic drive and windkessel vascular resistance in Phase 3 orthostasis. The clinical significance of this observation is mainly to raise awareness so that practitioners do appreciate the very important role of the natural central parasympathetic restraints of cardiovascular functions in maintaining sustainable and stable blood circulation in both healthy people and in patients.

Takeaway points

1. Degradation of the chronotropic function of the heart is a major event in Phase 3 orthostasis. It triggers exaggerated oscillations of both arterial pressure and heart rate at 0.1 Hz. Cardiovascular oscillation at this frequency is known as Traube–Hering–Mayer's waves present in all mammals irrespective of body size
2. Windkessel vascular resistance and chronotropic function of the heart are both at sub‐optimal levels that is insufficient for defending and preventing oscillations of arterial blood pressure during the severe cardiovascular stress in Phase 3 orthostasis
3. The drop in heart rate alone without effects on windkessel vascular resistance, CVT and CSB means the central sympathetic chronotropic drive to the heart (Zone C, Figure 3) can be, and is selectively degraded during Phase 3 orthostasis. Levels of CVT and CSB representing central parasympathetic restraints of cardiovascular control are kept at their lowest in Phase 3 comparable to that in Phase 2 orthostasis. This illustrates the flexibility provided by the organotopic neurons in CARS sites for independent adjustments of various individual functions during cardiovascular regulation
4. The natural central parasympathetic restraints play major roles in maintaining stable circulation of blood. These restraints can be quantified using validated clinical units. The average CVT level of 2.2 ± 0.5 LVS units and the average CSB level of 1.4 ± 0.3 ms.mmHg^−1^ are the lowest levels of central parasympathetic restraints that can sustain stable blood circulation during severe orthostatic stress in the population sample used here in this example of laboratory‐controlled progressive orthostatic stress

###### Orthostasis phase 4

**FIGURE 6:**
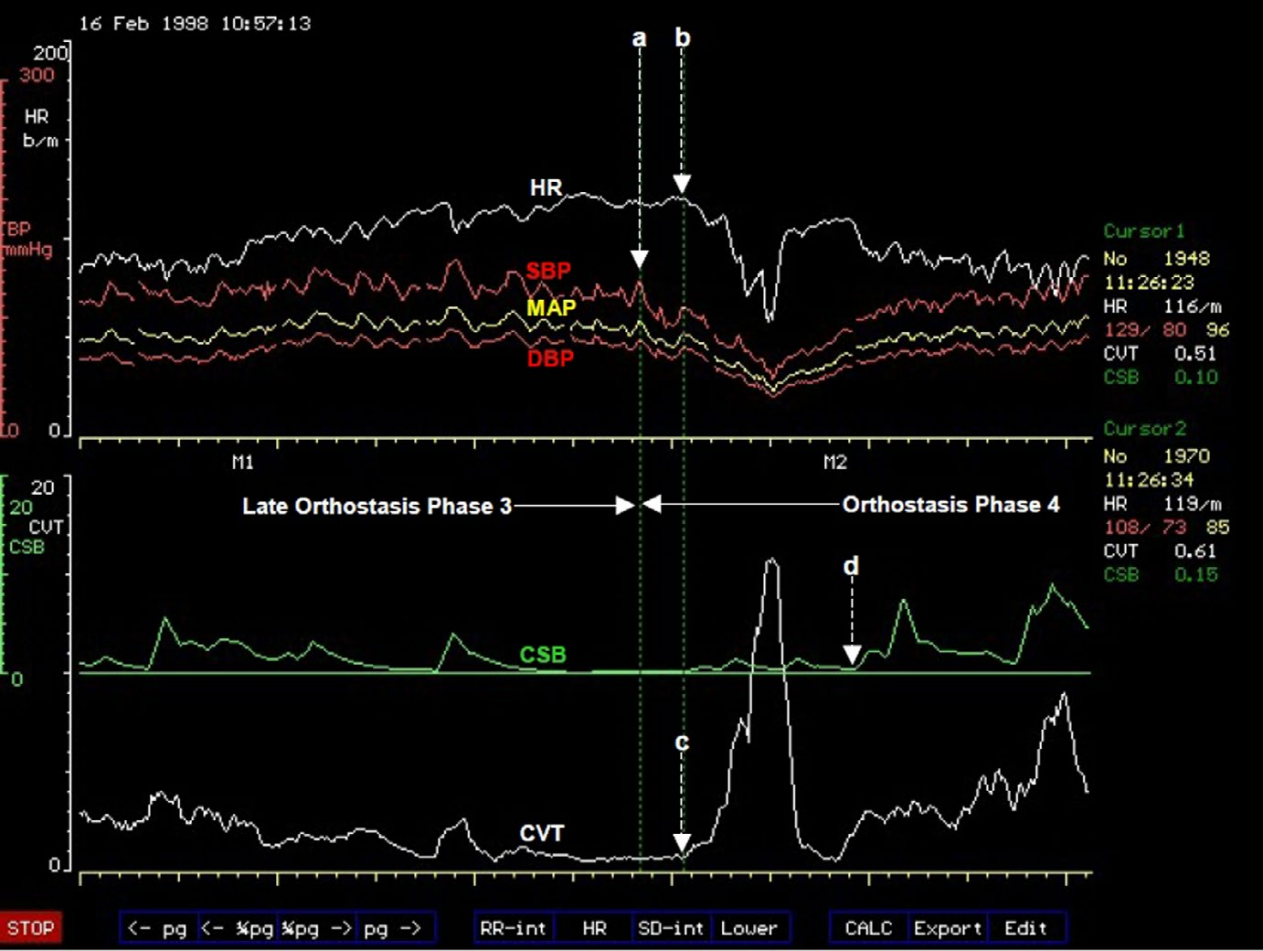
A computer screen capture from the NeuroScope displaying the following beat‐to‐beat traces of cardiovascular parameters at the transition from orthostasis Phase 3 to Phase 4. Heart rate (HR) expressed in beats.min^−1^; systolic (SBP), diastolic (DBP), and mean arterial blood pressures (MAP) measured in mmHg; cardiac sensitivity to baroreflex (CSB) measured in ms.mmHg^−1^ and cardiac vagal tone (CVT) measure in units of a linear vagal scale (LVS) (see text for descriptions and methods used to measure these cardiovascular parameters). Note the sudden and progressive decrease in blood pressure at the beginning of a vasodepressor response at (a). This was followed 11 s later by a progressive bradycardia starting at (b) in a cardiodepressor response. The bradycardia in the cardiodepressor response coincides with the beginning of a sudden rise of CVT at (c) during which CVT is reset from its very low level to an exaggerated high level. CSB was reset much later at (d) (see text for full explanation)

This is a phase of cardiovascular decompensation under severe orthostatic stress and it includes the resetting of central cardiovascular regulatory parameters to facilitate recovery from the cardiovascular decompensation. It starts with sudden decreases in arterial blood pressure and heart rate and is associated with intensification of symptoms of pre‐syncope including the loss of spatial awareness and may lead to postural syncope. The runaway drop in blood pressure precedes bradycardia by an average of 23 s in the majority of subjects (71%) in this laboratory‐controlled orthostatic stress and it is 11 s before the beginning of bradycardia in the illustration below (Figure 6). Reduction of forearm vascular resistance from the full cardiovascular compensation level of 170% to 137% of the supine baseline level represents a drop in windkessel vascular resistance (Zone D, Figure 3) to a level that can no longer defend and sustain a viable arterial blood pressure during the severe orthostatic stress in Phase 4 orthostasis. It therefore triggers a runaway fall in arterial blood pressure in Phase 4 orthostasis described as a vasodepressor response (Figure 6). The need for downgrading the level of windkessel vascular resistance is to try and compensate for the continual reduction of venous return (Zone D, Figure 3) caused by the combined effects of gravity and the continual LBNP being applied in this laboratory‐controlled example. Downgrading windkessel vascular resistance could be regarded as facilitation of arterial drainage into tissues to compensate for the reduced cardiac output caused by reduced venous return. The anterior insular cortex in CARS site 8 and the anterior Cingulate cortex in CARS site 6 (Figure 2), both are within the limbic system, and the premotor area of the frontal cortex in CARS site 11 would be the prominent supramedullary CARS sites (Figure 3) involved in the modulation and restraint of autonomic commands to the windkessel vessels to counteract the effects of the loss of venous return. This has been discussed before in detail in Functional Anatomy.

The collapse of arterial blood pressure during the vasodepressor response in Phase 4 orthostasis triggers a reset of the central restraint of the chronotropic function of the heart (Zone C, Figure 3) after a brief delay, leading to a rapid increase in the level of CVT (Figure 6). This rapid increase in the level of CVT coincides with a rapid bradycardia described as a cardiodepressor response in Phase 4 orthostasis (Figure 6). The rapid rise of CVT during the cardiodepressor response in Phase 4 orthostasis is withdrawn back to lower levels almost immediately once blood pressure recovery is initiated (Figure 6). The amygdala in CARS site 8 and the medial dorsal nucleus of the thalamus in CARS site 4, both are within the limbic system, and the periaqueductal grey matters in CARS site 7 in the midbrain (Figure 2) would be the prominent supramedullary CARS sites (Figure 3) in modulating the restraint of the chronotropic function of the heart in response to reduced venous return. This is discussed in more detail in Functional Anatomy. The reset and upgrade of CSB are not critical, therefore the timing of its execution is flexible during Phase 4 orthostasis. It can precede the CVT reset or lag behind it (Julu et al., 2003).

**FIGURE 7:**
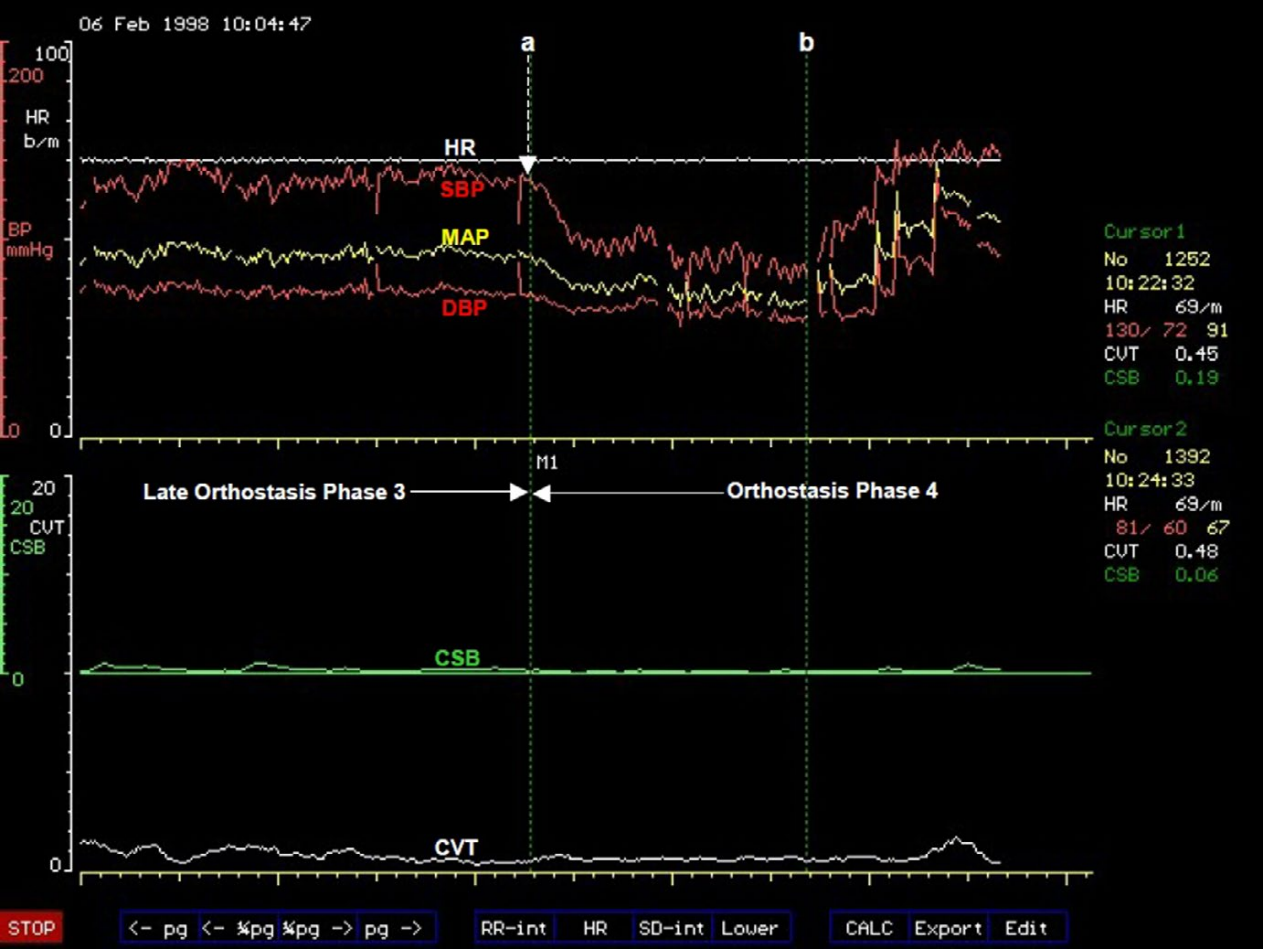
A computer screen capture from the NeuroScope displaying the following beat‐to‐beat traces of cardiovascular parameters at the transition from orthostasis Phase 3 to Phase 4 in a patient with a paced heart. Heart rate (HR) expressed in beats.min^−1^; systolic (SBP), diastolic (DBP) and mean (MAP) arterial blood pressures measured in mmHg; cardiac sensitivity to baroreflex (CSB) measured in ms.mmHg^−1^ and cardiac vagal tone (CVT) measure in units of a linear vagal scale (LVS) (see text for descriptions and methods used to measure these cardiovascular parameters). Note the blood pressure drop starting at (a) without any change in the paced heart rate in a vasodepressor response during tilt test associated with symptoms of pre‐syncope. The tilt test was abandoned at (b) to allow the blood pressure to recover

The rapid reset of CVT from its lowest to a high level in order to restrain the chronotropic function of the heart is essential for the initiation of blood pressure recovery in Phase 4 orthostasis. The clinical implication is that some practitioners use cardiac pacing to prevent the cardiodepressor response in Phase 4 orthostasis with the aims of treating postural syncope. This often fails to work because although a cardiac pacemaker can prevent the cardiodepressor response, it does not affect the vasodepressor response in Phase 4 orthostasis (Figure 7). Moreover, by preventing the cardiodepressor response during Phase 4 orthostasis, blood pressure recovery is abolished because cardiac pace makers artificially remove the natural means of initiating recovery and the blood pressure stays low (Figure 7). The patient remains symptomatic with evidence of deterioration of orthostatic tolerance with poor and delayed recovery following syncope.

Takeaway points

1. Progressive orthostatic stress induced by a combination of gravity and LBNP causes progressive decrease of the windkessel vascular resistance from the highest achievable level in Phase 1 to the lowest level at the beginning of Phase 4 orthostasis
2. Levels of windkessel vascular resistance less than 140% above the supine baseline level triggers vasodepressor response during orthostasis at low parasympathetic tone. Vasodepressor response is characterized by a runaway drop of arterial blood pressure associated with pre‐syncope symptoms. The drop in arterial blood pressure is independent of heart rate and occurs in people with paced hearts
3. Vasodepressor response in Phase 4 orthostasis triggers a rapid increase in CVT from its very low level after an average lag time of 23 s in majority of cases. The rapid increase in CVT coincides with a rapid decrease in heart rate described as a cardiodepressor response
4. The cardiodepressor response in Phase 4 orthostasis is characterized by a rapid and continuous decrease of heart rate. This can lead to asystole lasting below 5 s in less than 15% of cases
5. The rapid increase of CVT and the associated cardiodepressor response are essential for natural and quick recovery of arterial blood pressure from the vasodepressor response caused by orthostatic stress. Prevention of both CVT rise and the associated cardiodepressor response using cardiac pacing abolishes the natural and quick recovery from orthostatic vasodepressor response

#### 1.4.5 The physiological basis of postural orthostatic tachycardia

The international consensus statement on the definition of POTS is based on the magnitude of the tachycardia plus one other requirement of no change in arterial blood pressure that can be defined as postural hypotension. They also gave a definition of postural hypotension in this statement (Freeman et al., 2011). Our model (Figure 3) indicates that tachycardia, which represents the chronotropic function of the heart in the model, is just one of four independent cardiovascular functions that are regulated directly by organotopic neurons in the brain and in the brainstem (see Functional Anatomy) to defend arterial blood pressure. These cardiovascular functions shown in zones C and D (Figure 3) can be adjusted independently to defend the appropriate arterial blood pressure suitable for the prevailing physiological state. This was clearly illustrated during the laboratory‐controlled progressive orthostatic stress discussed above. It means if any one of these functions should fail, or is sub‐optimal, then the other three are adjusted accordingly to achieve the required blood pressure. This means the orthodox measurements of heart rate and blood pressure during orthostasis on their own will not give us any indication of possible pathophysiology of excessive tachycardia observed during clinical tests.

It is however clear from our model that both inotropic and chronotropic efforts of the heart are required to transform the venous return into cardiac output. These two cardiac functions are complementary to each other and are regulated independently by specific organotopic neurons in the caudal ventrolateral medulla (cVLM, see Functional Anatomy). It means either of the two functions can fail independently or any one of them can be used independently to compensate for failures of any other cardiovascular functions in zones C and D (Figure 3). This means excessive orthostatic tachycardia could be compensatory to poor or failing inotropic function of the heart, or failure of any other cardiovascular functions in zone D (Figure 3). It is also clear from the model that the extent of the arterial blood drainage away from the windkessel vessels regulated by the windkessel vascular resistance together with the cardiac output into the windkessel vessels determines the arterial blood pressure. Excessive orthostatic tachycardia could be compensatory to failure of either or both of these cardiovascular functions in zone D (Figure 3). We have used in our laboratory a combination of gravity and mechanical manipulation of venous vascular capacitance using LBNP to progressively change the level of venous return and mimic a few of the pathophysiology of POTS.

It is evident from the description of the physiology of orthostatic tachycardia using our model (Figure 3) that standardized and validated clinical methods for assessing individual cardiovascular functions in zones C and D in addition to the orthodox measurements of blood pressure and heart rate are required. There is also need to quantify if possible the central autonomic regulation of these cardiovascular functions. Advances in medical technology have enabled the quantification of central parasympathetic cardiovascular control and few other surrogate measures of the cardiovascular functions in zones C and D (Figure 3) and they are reviewed below.

### 1.5 Recommended neurophysiological assessments in a clinical laboratory to identify the pathophysiology of postural orthostatic tachycardia syndrome

#### 1.5.1 Autonomic target‐organs neurophysiological tests (ATONT)

##### Definition of autonomic failure

The phrase “Autonomic Failure" when used as a clinical term means the inability of the autonomic nervous system to execute co‐ordinated and standardized autonomic tasks that have been identified and validated for clinical examinations of the system. The autonomic tasks are set up in the form of special maneuvers recommended for clinical tests of autonomic function by an International Committee in a consensus statement of San Antonio (Kahn, 1988). Diagnosis of autonomic failure cannot be based on a single abnormal test parameter because the autonomic nervous system either influences or controls the functions of all the organs in the body. It requires a set of carefully designed clinical maneuvers aimed to examine functions of specific target‐organs in order to draw conclusions whether or not such target‐organs have normal functions. We have designed and validated multiple parameters described below. Some of these are derived from the internationally recommended maneuvers mentioned above and some are improvements of the recommendations, but we have designed some entirely in our laboratory to diagnose and characterize autonomic failures according to the specific target‐organs we intend to examine. We would report a failure or dysfunction of a target‐organ if its function lies outside the range of our normal values. Our laboratory standards are based on 250 healthy subjects aged between 13 and 79 years.

##### Assessment of central parasympathetic function

###### Resting supine cardiac vagal tone (CVT)

**FIGURE 8:**
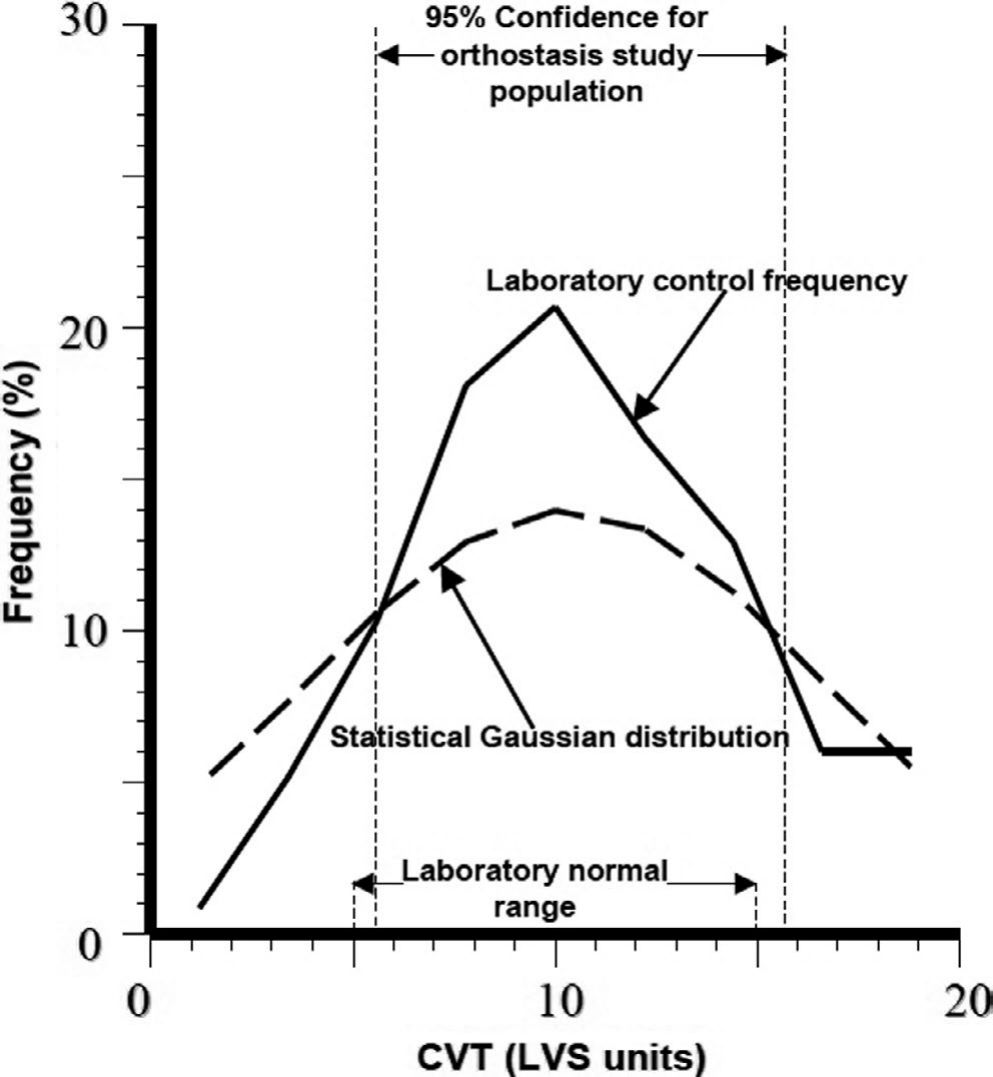
A frequency distribution plot of the observed values of cardiac vagal tone (CVT) measured in units of a linear vagal scale (LVS) at rest in supine position in the laboratory control population (*n* = 250, see text for details). The theoretical statistical Gaussian distribution with the same population mean and standard deviation is superimposed in this plot. Chi squared test showed a normal distribution of CVT in our laboratory control population. The laboratory normal range of resting supine values of CVT is indicated in the plot together with the 95% confidence interval of baseline CVT values in a sample population used in the study of the effects of progressive orthostatic stress on autonomic regulation of the cardiovascular system (see text for details)

This is a measure of the central parasympathetic restraint of the chronotropic function of the heart (Zone C, Figure 3) that comes through the vagus nerve (A, Figure 3). It is measured continuously on beat‐to‐beat basis as “pulse‐synchronized phase shifts in consecutive cardiac cycles” using the NeuroScope (Medifit Instruments Ltd, London, UK) in a quiet room at controlled room temperature of 21 ± 1°C. It is essentially a form of jitter that is quantified continuously from the electrocardiogram (ECG) using a circuit of electronic integrators and phase detector to convert the pulse interval jitters into voltages (Little, Julu, Hansen, & Reid, 1999), which are then calibrated into units of the atropine‐derived linear vagal scale (LVS (Julu, 1992)). The least value in the units of LVS is zero, equivalent to full atropinization of human subjects (Julu, 1992). The LVS is therefore a validated clinical unit of cardiac vagal tone with an absolute zero reference point. The frequency distribution of CVT measured in units of LVS in our laboratory control population is approximately Gaussian (Figure 8).

###### Respiratory gating of cardiac vagal tone (CVT) In supine position

**FIGURE 9:**
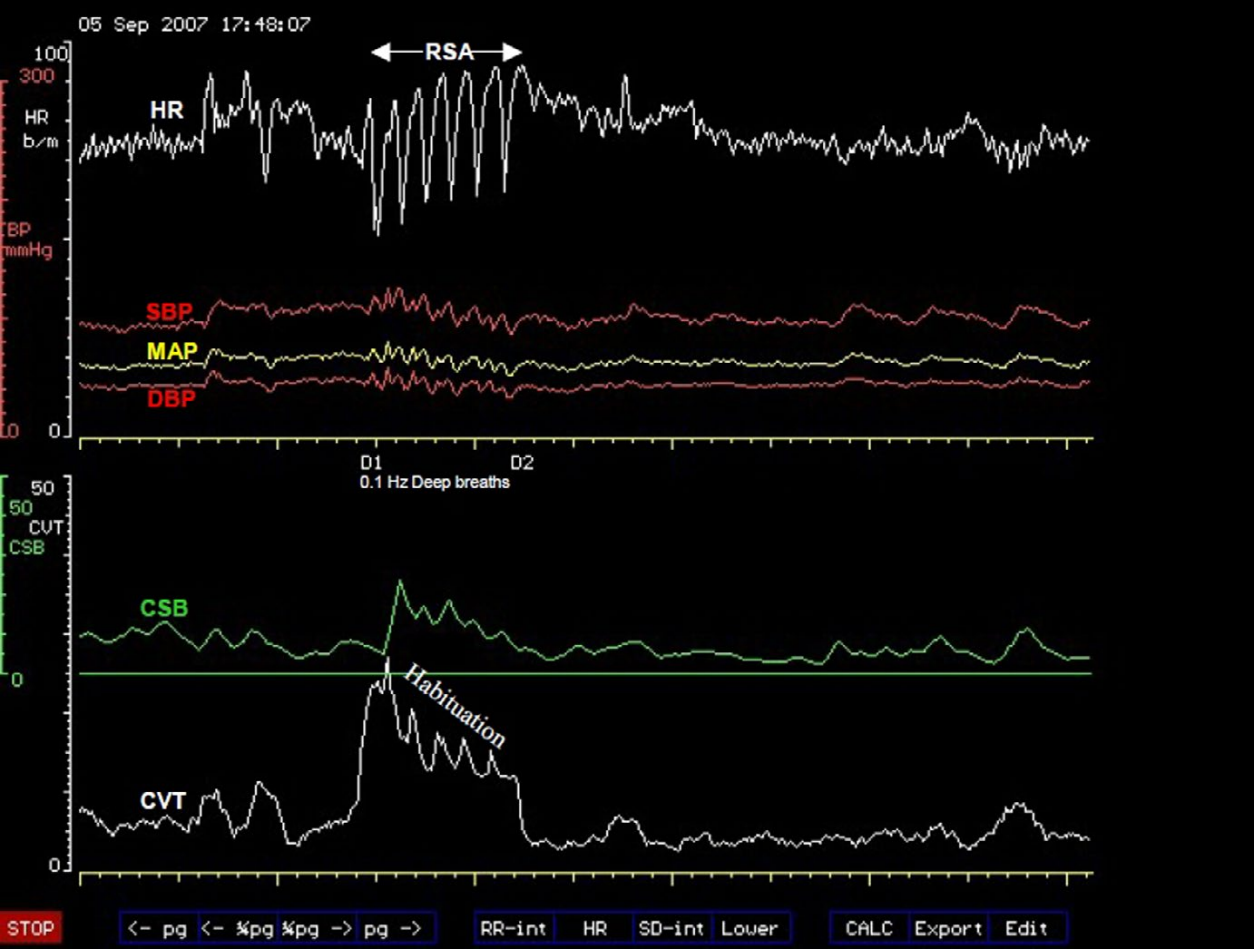
A computer screen capture from the NeuroScope displaying the following beat‐to‐beat traces of cardiovascular parameters during slow deep breathing at 0.1 Hz. Heart rate (HR) expressed in beats.min^−1^; systolic (SBP), diastolic (DBP) and mean (MAP) arterial blood pressures measured in mmHg; cardiac sensitivity to baroreflex (CSB) measured in ms.mmHg^−1^ and cardiac vagal tone (CVT) measure in units of a linear vagal scale (LVS) (see text for descriptions and methods used to measure these cardiovascular parameters). Note the sharp rise in CVT coinciding with the first deep breath and displaying clear respiratory modulation and habituation during subsequent deep breaths. This is associated with respiratory sinus arrhythmia (RSA) with the HR oscillating at 0.1 Hz

The central parasympathetic tone with inherent cardiac pulse and breathing rhythms and responds to voluntary changes in breathing rhythms is also known as cardiac vagal tone and it is generated by cardiac vagal premotor neurons (cVPN) in the nucleus ambiguus (NA) in the brainstem as discussed previously in Functional Anatomy. The magnitude of respiratory gating of CVT at the level of cVPN in NA can be quantified (Figure 9) and used to assess the integrity of cardiorespiratory neurons within the brainstem. This is a central cardiorespiratory cholinergic function of the brainstem at the level of cVPN (Gilbey et al., 1984). Lying in supine position, the subject is asked to take a deep breath lasting 4 s and to breathe out forcefully and reach up to expiratory reserve volume within 6 s and repeating this exercise six times while CVT is being measured continuously (Figure 9) using the NeuroScope (Medifit Instruments Ltd, London, UK) in a quiet room with controlled room temperature as described above. Deep breathing at 0.1 Hz should increase CVT from baseline (Figure 9) by values ranging between 5 and 15 LVS units and should show clear respiratory modulations (Figure 9) in the continuous CVT record at the same frequency (Our laboratory standard).

###### Resting Supine Cardiac Sensitivity to Baroreflex (CSB)

This is a measure of the beat‐to‐beat baroreflex central sympathetic restraint exerted directly within the brainstem (see Functional Anatomy). It is a measure of the well‐known baroreflex restraint of sympathetic activity, mainly the vasomotor tone in the RVLM, and the baroreflex restraint is coming through the NTS (Dampney, 2009; Schreihofer & Guyenet, 2002). The resting supine CSB is measured using the NeuroScope (MediFit Instruments Ltd, London) as previously described (Julu et al., 2003) in a quiet room at controlled room temperature as described above. The index is defined as the increase in pulse interval per unit increase in systolic pressure and it is measured in units of ms.mmHg^−1^. It is a beat‐to‐beat baroreflex negative feedback control of arterial blood pressure. The least value of CSB is zero representing no negative feedback cardiovascular control at all. The normal range in our laboratory population is between 6 and 12 ms.mmHg^−1^. The 95% confidence interval in the population sample used in the progressive orthostatic stress study described here in this review is between 5 and 11 ms.mmHg^−1^ similar to our laboratory normal range. The method allows the detection of rapid changes in CSB within a continuous measurement, which represents real‐time engagements and disengagements of the baroreflex negative feedback cardiovascular control. Disengagement of negative feedback cardiovascular control indicates cardiovascular arousal in preparation for imminent changes in either blood pressure alone, or heart rate alone (Figure 1), or both heart rate and blood pressure at the same time (Figure 1).

###### Assessment of central baroreflex gain

**FIGURE 10:**
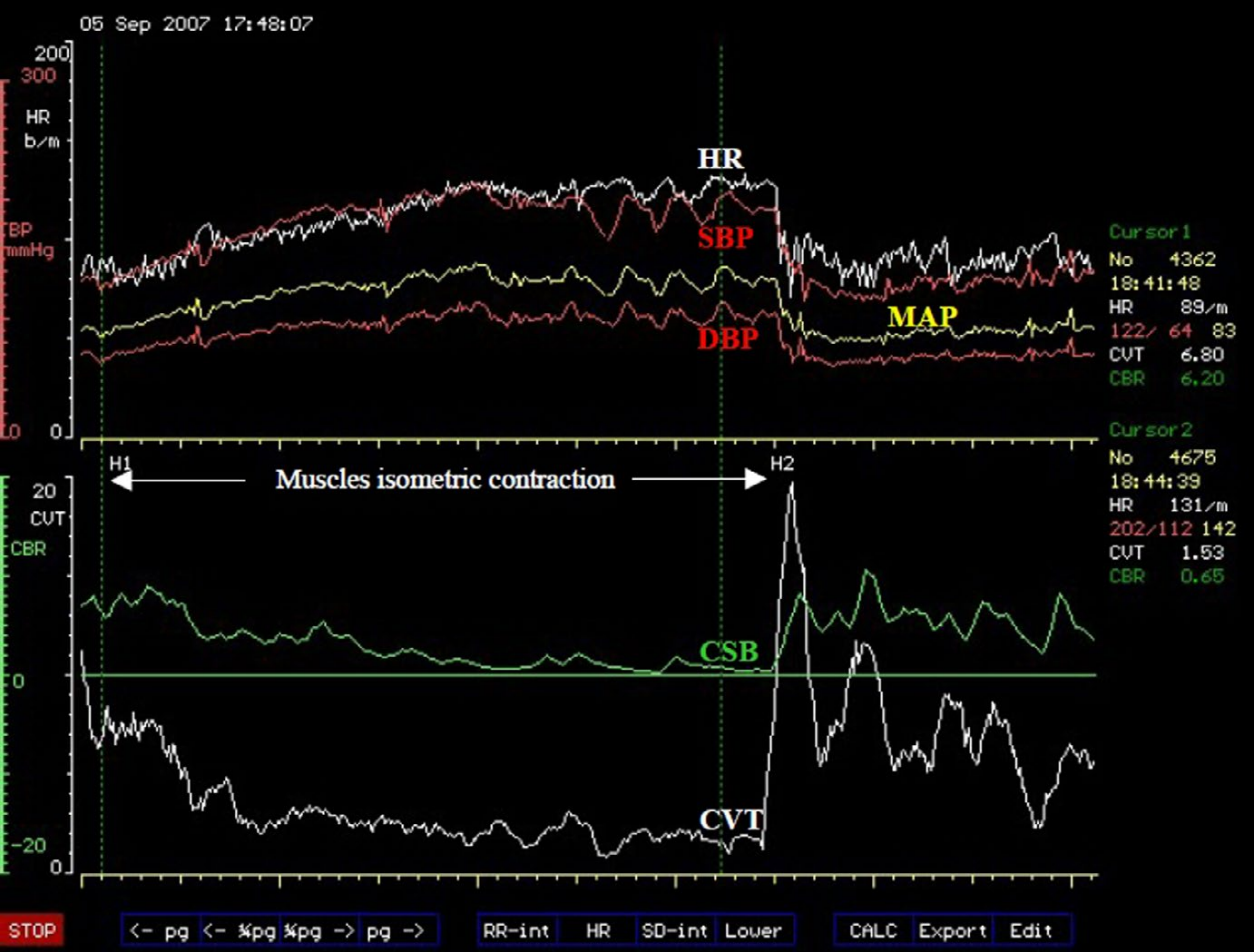
A computer screen capture from the NeuroScope displaying the following beat‐to‐beat traces of cardiovascular parameters during sustained isometric contraction of skeletal muscles of the dominant hand for 3 min. Heart rate (HR) expressed in beats.min^−1^; systolic (SBP), diastolic (DBP) and mean (MAP) arterial blood pressures measured in mmHg; cardiac sensitivity to baroreflex (CSB) measured in ms.mmHg^−1^ and cardiac vagal tone (CVT) measure in units of a linear vagal scale (LVS) (see text for descriptions and methods used to measure these cardiovascular parameters). Note the withdrawal of both CVT and CSB early at the beginning of muscle contraction, which starts at H1 and the levels are maintained at minimum until the end of muscle contraction at H2. This is accompanied by sustained and concurrent increases of both HR and all the arterial blood pressures. There was abrupt and exaggerated reset of both CVT and CSB to very high levels at the end of muscle contraction at H2 associated with oscillations and immediate and sharp decreases of heart rate and blood pressures back to baseline pre‐exercise levels

We use the isometric contraction of the skeletal muscles of the dominant hand during a sustained handgrip at 50% of the maximum sustainable force measured in Newton (N) for a target duration of three minutes to reduce and keep the baroreflex negative feedback cardiovascular restraint to its lowest level (Figure 10). Withdrawal of the baroreflex negative feedback control measured as CSB in the upright‐seated position is achieved within 1 min of sustained handgrip and stays at its least level for the remaining duration of the handgrip. This is confirmed visually on the computer screen during the test procedure (Figure 10). Since CSB is at its lowest level, the gradients of the concurrent slow and sustained rise in heart rate and arterial blood pressure during the second and third minute of sustained handgrip (Figure 10) are regulated by the supramedullary CARS sites (Figure 3). The periorbital frontal lobes in CARS site 9 (Figure 2) are the dominant bilateral supramedullary CARS sites during the restraints of both heart rate and arterial blood pressure during isometric contraction of skeletal muscles as previously discussed in Functional Anatomy. The gradient of concurrent changes in heart rate and arterial blood pressure is a measure of baroreflex gain and in this case it is a supramedullary central gain. We define this central baroreflex responsiveness (BRR) or gain as the absolute value of the ratio ΔR‐R/ΔSBP, where ΔR‐R is the change in the electrocardiographic (ECG) R‐R interval, associated and concurrent with ΔSBP the change in systolic BP from baseline to the respective levels at the end of three minutes of sustained handgrip. The central baroreflex gain can be predicted from a mathematical equation (Julu, Hansen & Jamal, 1996): BRR = 2.42 × 10^(3h‐5)^ ms.mmHg^−1^, where h is the subject's height in meters, or can be expressed in terms of the natural number “e” where BRR = 6.35 × 10^(eh‐5)^ ms.mmHg^−1^. The observed value from the ratio ΔR‐R/ΔSBP should be within 50% of the predicted value in the age group 15–79 years (Our laboratory standard). The ECG R‐R intervals, CSB and the arterial blood pressure are monitored and measured continuously (Figure 10) using the NeuroScope during this assessment of the central baroreflex gain (Figure 12).

##### Assessment of Peripheral baroreflex function

###### Mechanical digital massage of the carotid sinus

**FIGURE 11:**
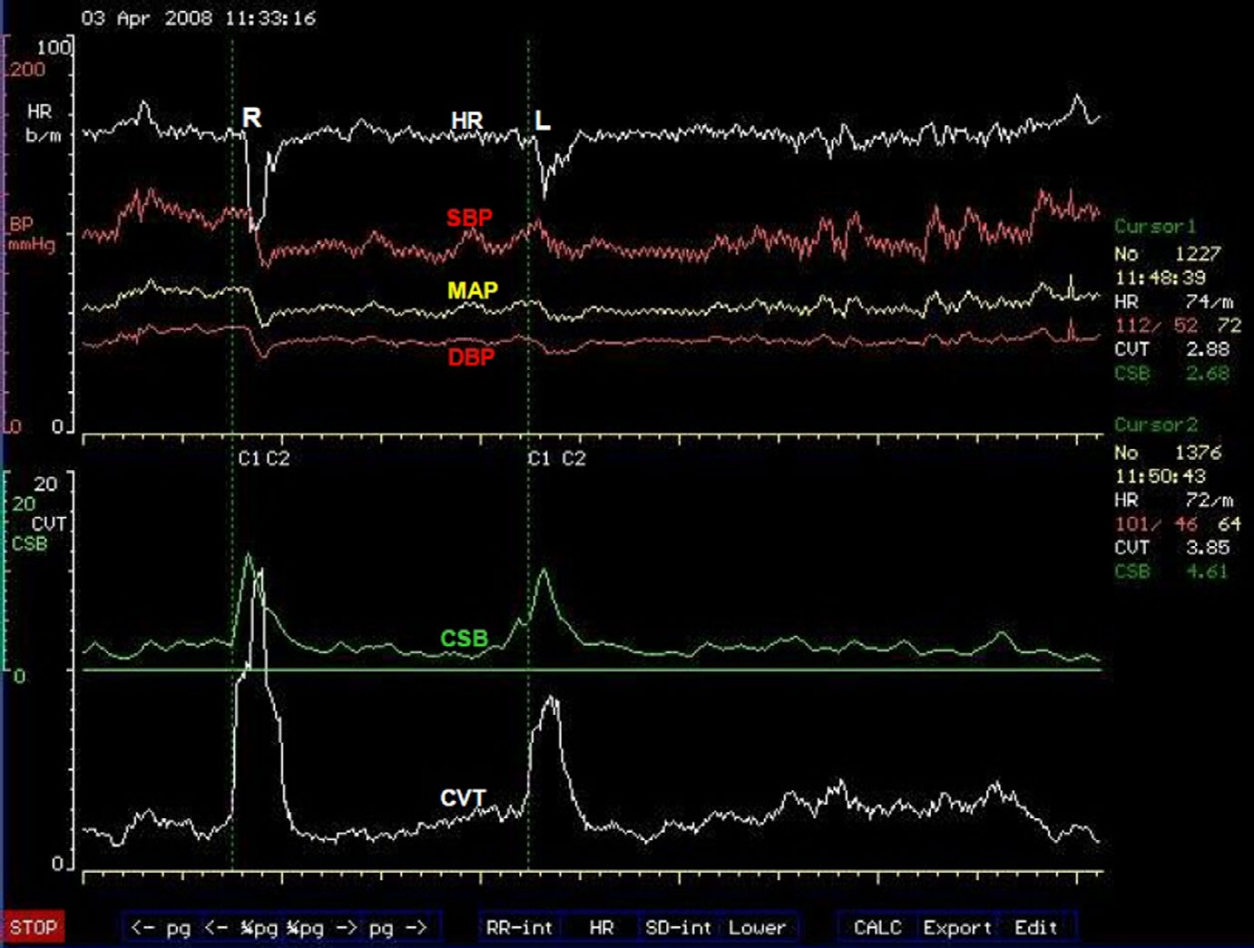
A computer screen capture from the NeuroScope displaying the following beat‐to‐beat traces of cardiovascular parameters during two episodes of mechanical digital massages of the two carotid sinuses. Heart rate (HR) expressed in beats.min^−1^; systolic (SBP), diastolic (DBP) and mean (MAP) arterial blood pressures measured in mmHg; cardiac sensitivity to baroreflex (CSB) measured in ms.mmHg^−1^ and cardiac vagal tone (CVT) measure in units of a linear vagal scale (LVS) (see text for descriptions and methods used to measure these cardiovascular parameters). The right carotid sinus was massaged for 15 s starting from C1 to C2 at R. Note the sharp increases in both CVT and CSB from baseline levels accompanied by sharp drops in both HR and arterial blood pressures. The procedure is repeated on the left carotid sinus at L with similar increases in CVT and CSB accompanied by sharp drops in HR and arterial blood pressures.

**FIGURE 12:**
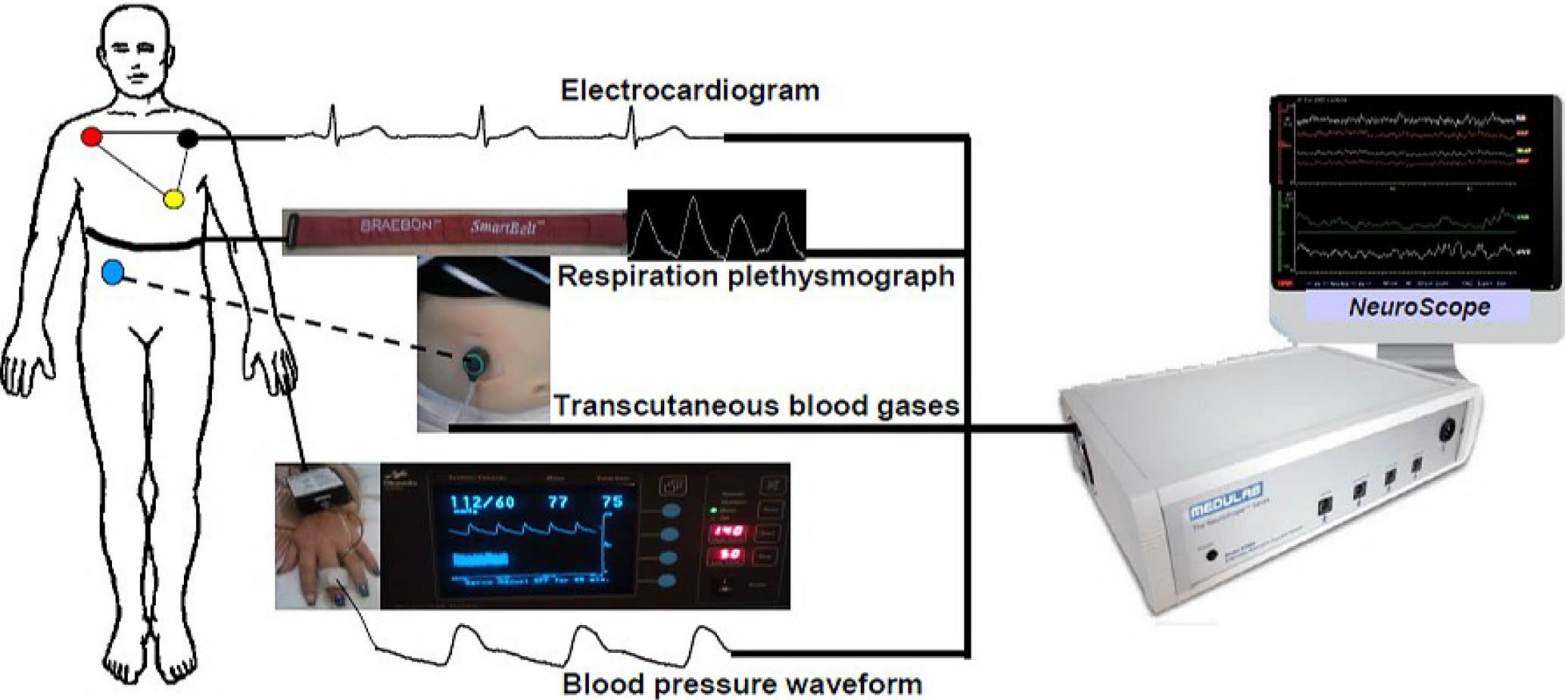
A schematic representation of non‐invasive continuous streams of biometrics obtained from the human body by the NeuroScope during the assessment of autonomic functions. Continuous electrocardiogram (ECG) is recorded using three chest‐electrodes in a modified Einthoven’s triangle, but configured within the NeuroScope as Einthoven's ECG lead II. Continuous stream of breathing movements is recorded using respiration impedance plethysmography (RIP), which is coupled within the NeuroScope to facilitate the discrimination and identification of apneusis, apnea, Valsalva's maneuvers and other respiratory dysrhythmias. The respiration plethysmogram belt is applied around the chest at the xiphisternal level to capture both thoracic and upper abdominal movements. Transcutaneous blood gases are recorded continuously during autonomic assessment using the dual oxygen and carbon dioxide electrode (Radiometer, Copenhagen). The transcutaneous blood gases electrode measures tension of gases in tissues, not gas tension in the bloodstream. The tension of gases in the tissues correlates with local capillary blood flow, tissue metabolism and temperature, all of which are influenced by the autonomic nervous system and form part of autonomic functions. Tissue temperature in the surrounding of the transcutaneous blood gases sensor is kept constant using a heating element within the sensor. In our practice, the sensor is placed sub‐costal in the mid clavicular line close to the liver where it is relatively warmer than other peripheral sites. Continuous stream of arterial blood pressure waveform is recorded from the finger using photoplethysmography and Penáz's principle of arterial pressure unloading through volume‐clamp (Finapres Medical Systems, Enschede the Netherlands). All biometrics are synchronized to the nearest millisecond, date and time‐stamped before they are used to derive cardiovascular and autonomic parameters using the VaguSoft software (Medifit Instruments Ltd, London).

Carotid massage using digital pressure applied on the carotid sinus in a resting supine position at the rate of the subject's own heartbeats for the duration of 15 s should cause an increase in the CVT (Figure 11), usually of the magnitudes between 5 and 20 LVS units above the baseline level in the age group 15–79 years (our laboratory standard). We describe this as a standardized clinical measure of cardiodepressor response of peripheral baroreflex accompanied by a sharp drop in the heart rate (Figure 11). The same carotid massage should cause a drop of systolic arterial blood pressure by at least 10 mmHg (Figure 11), but should not exceed 30 mmHg (our laboratory standard). The CVT and the arterial blood pressure are monitored and measured continuously (Figure 11) using the NeuroScope (Figure 12) during this assessment of the baroreflex function.

###### Pharmacological peripheral baroreceptor excitation

Intravenous injection of phenylephrine or other α_1_‐adrenergic vasoactive drugs in a resting supine position should cause a concurrent rise in both blood pressure and ECG R‐R intervals and the two should be statistically associated with a gradient of R‐R intervals rise per systolic blood pressure rise between 5 and 12 ms.mmHg^−1^ (similar to the normal range of the resting supine CSB described above) and a correlation coefficient greater than 0.9 (Our laboratory standard). The ECG R‐R intervals and the arterial blood pressure are monitored and measured continuously using the NeuroScope (Figure 12) during this assessment of peripheral baroreflex function.

##### Assessment of sympathetic α*_1_‐Adrenergic Denervation Hypersensitivity*

A small intravenous bolus dose of 25 μg of phenylephrine is used to test for denervation hypersensitivity of sympathetic α_1_‐adrenergic nerves. This will cause systolic blood pressure rise of more than 15 mmHg in denervation hypersensitivity (our laboratory standard). All the arterial blood pressures; SBP, DBP and MAP, including the parasympathetic restraints; CSB and CVT and the heart rate are monitored and measured continuously during this test using the NeuroScope (Figure 12).

##### Assessment of sympathetic control of cardioaccelerator function

We use the isometric contraction of the skeletal muscles of the dominant hand during a sustained handgrip at 50% of the maximum sustainable force measured in Newton (N) for the duration of 3 min to reduce and keep the baroreflex negative feedback cardiovascular restraint and the central parasympathetic tone to their lowest levels (Figure 10). Withdrawals of both the baroreflex negative feedback control measured as CSB and the central parasympathetic activity measured as CVT within the first minute of sustained handgrip mean that the continual increase in heart rate during the second and third minute of sustained handgrip (Figure 10) represents the central sympathetic drive of cardioaccelerator function (Pathway B, Figure 3) with minimal parasympathetic restraint (Pathway A, Figure 3; see also Functional Anatomy). The heart rate should increase above the resting pre‐exercise value (before H1, Figure 10) by 22% to 45% at the end of 3 min of sustained handgrip (at H2, Figure 10) in subjects aged 15–79 years (Our laboratory standard). Beat‐to‐beat heart rate, the arterial blood pressures; SBP, DBP and MAP, including the parasympathetic restraints; CSB and CVT are monitored and measured continuously (Figure 10) using the NeuroScope during this test (Figure 12).

##### Assessment of sympathetic vasomotor control of windkessel vascular resistance

We use the isometric contraction of the skeletal muscles of the dominant hand described above to reduce and keep the baroreflex negative feedback cardiovascular restraint and the central parasympathetic tone to their lowest levels in this procedure. The continual increase in diastolic blood pressure (DBP) during the second and third minute of sustained handgrip represents the central sympathetic vasomotor drive of the windkessel vascular resistance (Zone D, Figure 3) with minimal parasympathetic restraint (see full discussion in Functional Anatomy). We use the rising DBP during sustained handgrip as a surrogate of increasing central sympathetic vasomotor drive to windkessel vascular resistance because DBP has a linear relationship with muscles sympathetic nerve activity during this procedure (Halliwill, Taylor, & Eckberg, 1996). Diastolic arterial blood pressure is a function of the windkessel vascular resistance (Westerhof, Lankhaar, & Westerhof, 2009). The skeletal muscle vascular bed provides most of the windkessel vascular resistance during muscle contraction because the sympathetic drive to skin is significantly inhibited (see Functional Anatomy above) and sympathetic drive to the splanchnic vascular bed is mainly to regulate venous vascular capacitance (Greenway, 1983) out of the three major vascular beds used for arterial blood pressure control (Figure 2 and Figure 3). The diastolic blood pressure should increase above the resting pre‐exercise level (before H1, Figure 10) by no less than 15 mmHg in all adults (Ewing, Irving, Kerr, Wildsmith, & Clarke, 1974) at the end of 3 min (at H2, Figure 10). Our laboratory range for the age range 15–79 years is an increase above the pre‐exercise level by 18–46 mmHg at the end of three minutes of sustained handgrip at 50% of the maximum sustainable force measured in Newton. All the arterial blood pressures; SBP, DBP and MAP, including the residual parasympathetic restraints; CSB and CVT and the heart rate are monitored and measured continuously (Figure 10) during this test using the NeuroScope (Figure 12).

##### Assessment of sympathetic control of venous vascular capacitance in the splanchnic region

We use a positive intra‐thoracic pressure of 40 mmHg lasting 15 s during Valsalva's maneuver to suddenly reduce venous return to the heart (Zone D, Figure 3) and provoke an auto‐transfusion response (Lundgren, 1983). The auto‐transfusion is caused by a neurogenic constriction of vessels and organs in the splanchnic region. In order to optimize the assessment of venous return from the splanchnic region, the Valsalva's maneuver is performed in the upright‐seated position with the subject stooping forward so that the inguinal ligaments compress the iliac veins to eliminate most of the venous return from the lower limbs. Before the auto‐transfusion response, arterial blood pressure increases suddenly in a Phase I response, then it starts to fall rapidly due to lack of venous return in the early part of Phase IIe. The trough of SBP in Phase IIe should be within 25 mmHg from the pre‐maneuver baseline level to indicate adequate and normal venous return during the positive intra‐thoracic pressure (Our laboratory standard). Phase IIe is arrested at the beginning of the auto‐transfusion response, which starts to restore arterial blood pressure back toward the pre‐maneuver level during the positive intra‐thoracic pressure in the late Phase IIi. This should end with SBP at or not more than 15 mmHg above the pre‐maneuver baseline level to indicate adequate auto‐transfusion response (Our laboratory standard). Abrupt end of the positive intra‐thoracic pressure of Valsalva's maneuver reverses the pressure from positive to negative intra‐thoracic creating a suction effect in Phase III, which decreases the arterial blood pressure suddenly and the SBP at the trough of Phase III should be within 25 mmHg from the pre‐maneuver baseline level to indicate sufficient venous blood volume during negative intra‐thoracic suction following the auto‐transfusion response (Our laboratory standard). All the arterial blood pressures; SBP, DBP, and MAP, including the parasympathetic restraints; CSB and CVT and the heart rate are monitored and measured continuously during this test using the NeuroScope (Figure 12).

## CONFLICT OF INTEREST

I am the inventor of the NeuroScope, but the machine is patented by the University of Glasgow in the United Kingdom. The University sold the sole right of exploitation of the patent to Medifit Instruments Limited, London, the United Kingdom. I have no financial connection with this company but sometimes give them scientific advice without pay.
